# Ibrutinib induces chromatin reorganisation of chronic lymphocytic leukaemia cells

**DOI:** 10.1038/s41389-019-0142-2

**Published:** 2019-05-10

**Authors:** Katie B. Holmes, Ildar I. Sadreev, Andy C. Rawstron, Tal Munir, David R. Westhead, Peter Hillmen, Pascal F. Lefevre

**Affiliations:** 1 Section of Experimental Haematology, Leeds Institute of Medical Research at St James’s, University of Leeds, Leeds, UK; 2 Bioinformatics Group, Institute of Molecular and Cellular Biology, University of Leeds, Leeds, UK; 3 Haematological Malignancy Diagnostic Service (HMDS), St. James’s Institute of Oncology, Bexley Wing Beckett Street, Leeds, LS9 7TF UK

**Keywords:** Histone post-translational modifications, Targeted therapies

## Abstract

Chronic lymphocytic leukaemia (CLL) is the most common leukaemia in Western countries. It has recently been shown that the homogeneity of the chromatin landscape between CLL cells contrasts with the important observed genetic heterogeneity of the disease. To gain further insight into the consequences of disease evolution on the epigenome’s plasticity, we monitored changes in chromatin structure occurring in vivo in CLL cells from patients receiving continuous Ibrutinib treatment. Ibrutinib, an oral inhibitor of the Bruton’s tyrosine kinase (BTK) has proved to be remarkably efficient against treatment naïve (TN), heavily pre-treated and high-risk chronic lymphocytic leukaemia (CLL), with limited adverse events. We established that the chromatin landscape is significantly and globally affected in response to Ibrutinib. However, we observed that prior to treatment, CLL cells show qualitative and quantitative variations in chromatin structure correlated with both EZH2 protein level and cellular response to external stimuli. Then, under prolonged exposure to Ibrutinib, a loss of the two marks associated with lysine 27 (acetylation and trimethylation) was observed. Altogether, these data indicate that the epigenome of CLL cells from the peripheral blood change dynamically in response to stimuli and suggest that these cells might adapt to the Ibrutinib “hit” in a process leading toward a possible reduced sensitivity to treatment.

## Introduction

Chronic lymphocytic leukaemia (CLL) originates from clonal proliferating B-cells with patients mainly presenting with lymphadenopathy, splenomegaly, and lymphocytosis^1^. A combination of fludarabine, cyclophosphamide and rituximab (FCR) represents the standard therapy^2^. However, a majority of patients relapse with most of them eventually succumbing to CLL. Encouraging results of several forerunner clinical trials that target the activity of PI3Kδ, BTK or SYK, highlight the therapeutic potential of inhibiting BCR signalling^3,4^. Ibrutinib (PCI-32765), a specific and irreversible inhibitor of Bruton’s Tyrosine Kinase (BTK), is a small molecule orally administered, providing a selective and irreversible inhibition of BTK. In extensive studies, Ibrutinib has shown extremely promising results in front-line treatment as well as in relapsed/refractory (RR) CLLs^5,6^ and is now tested in combination with other molecules^7^. However, cases of drug resistance have emerged^8,9^.

In recent years, a large body of work has highlighted the complexity of the regulatory mechanisms controlling gene expression by external environmental stimuli and signalling pathways for which chromatin plays a central role. The eukaryotic genomes are partitioned into functionally autonomous clusters in which gene expression is either positively or negatively controlled. In active clusters, promoters are highly enriched for the histone lysine 4 trimethylation mark (H3K4me3), whereas activated enhancers display enrichment of histone H3 lysine 4 mono-methylation and di-methylation (H3K4me1/2) and lysine 27 acetylation (H3K27ac). The equilibrium between open and repressed chromatin is dynamic and can change transiently or permanently in response to various endogenous or exogenous stimuli. These processes are controlled by several classes of epigenetic factors. One such class of key epigenetic regulators are the polycomb group (PcG) proteins, which are a family of transcriptional repressors, primarily known in maintaining cell identity, but also implicated in the control of cellular proliferation and neoplastic development^10–12^. A recent study has shown that the lack of transcription triggers deposition of H3K27me3, the repressive mark mediated by the polycomb-repressive complex 2 (PRC2)^13^. The core PRC2 complex comprises of four components, its enzymatic subunit with methyltransferase activity EZH1 or EZH2, SUZ12, EED and RbAp46/48. Furthermore, bivalent promoters, which harbour both active and silent marks (H3K4me3, H3K27me3), are usually CpG rich^14^. They have been mainly identified in stem cells, but can persist during differentiation as seen in T and B cells^15^.

EZH2 expression is correlated with proliferation to oppose cell division-mediated dilution of H3K27me3^16^. In B cells, EZH2 is highly expressed in lymphoid progenitors and is necessary for early lymphopoiesis^17^. EZH2 declines in mature resting B cells but is transiently reactivated in the germinal centre where dividing Ki67+ centroblasts are associated with its expression^18,19^. EZH2 is required for the formation and function of the germinal centre, where it participates to the establishment of bivalency at key regulatory promoters to transiently block differentiation^15^.

A recent study proposed an extensive epigenomic characterisation of CLL cells, which indicated that if DNA methylation or chromatin accessibility shows patterns characteristic of the cellular origin of these cells, active chromatin marks like H3K27ac follow other more complex dynamics^20^. To further assess the correlation between chromatin organisation and the evolution of the disease, we analysed the plasticity of the chromatin landscape of CLL cells from patients treated with Ibrutinib.

Our analysis revealed that the CLL cell populations in the peripheral blood was heterogeneous, including cells with various proportions of epigenomic traits characteristic of activated B cells. Moreover, the initial chromatin remodelling in response to Ibrutinib was dependent upon these initial traits but was converging toward a similar organisation after few months. Prolonged exposure to Ibrutinib, inducing forced maintenance of cells in G0 phase, was associated with a complete loss of H3K27 post-translational modifications including the polycomb-dependent repressive mark. This work suggests that CLL cells lose some epigenetic constraints in response to treatment.

## Results

### Prolonged Ibrutinib treatment correlates with loss of histone H3K27 marks

**Fig. 1:**
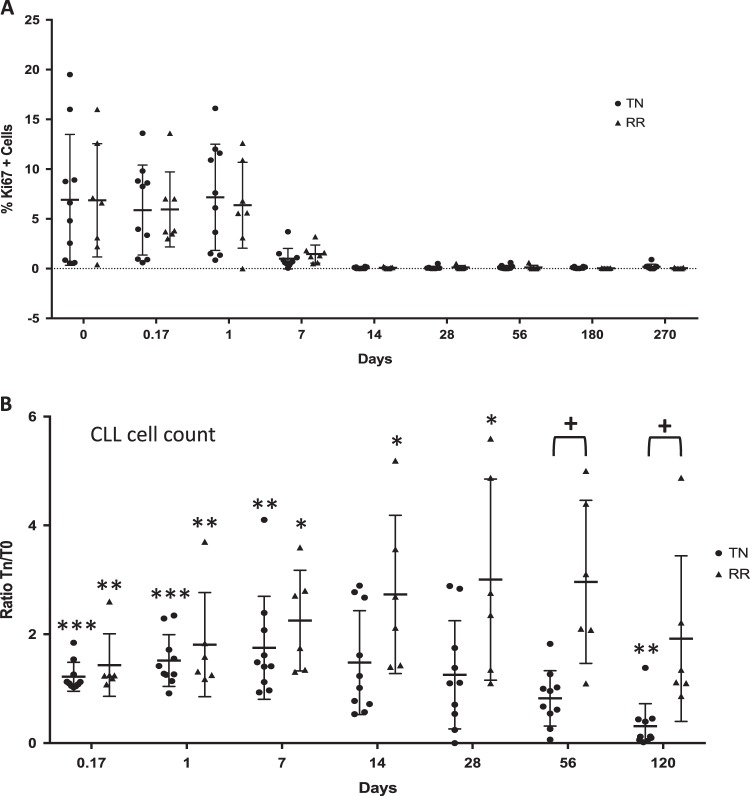
Ibrutinib treatment induces lymphocytosis in CLL cells. **a** Evolution of CLL cells count and (B) Ki67+ cells for the 16 patients included in the study comparing treatment-naïve (TN) and relapsed-refractory (RR) patients during (**a**) the first 120 days or (**b**) up to 9 months on Ibrutinib. Peripheral CLL cell counts from peripheral blood are expressed as a ratio vs. cell count the day of the first Ibrutinib ingestion. Paired Student’s *t*-test has been performed comparing cell count at variable time points compared to T0, *t-value < 0.05, **t-value < 0.01 and ***t-value < 0.001. Student’s *t*-test has also been performed comparing TN to RR for each time point, ^**+**^*t*-value < 0.05

The IcICLLe trial is a feasibility study investigating the mechanism of action of Ibrutinib. Patients received continuous oral therapy with Ibrutinib (420 mg OD) and CLL cells were collected from the peripheral blood before and at regular time points during treatment (Supplementary Table 1). The percentage of Ki67-positive cells in the blood increased in the first 24 h after Ibrutinib treatment initiation but then dropped rapidly to completely disappear after 14 days in all patients (Fig. 1a). At the same time, an important and transient increase in CLL cell count, characteristic of Ibrutinib-dependent lymphocytosis, occurred within the first hours after the start of the treatment and fall back significantly to its initial level from 14 days and 2 months for treatment naïve (TN) and RR patients, respectively (Fig. 1b). This result is consistent with previously described Ibrutinib-dependent rapid reduction of lymphadenopathy accompanied by transient lymphocytosis in CLL^21^.

**Fig. 2:**
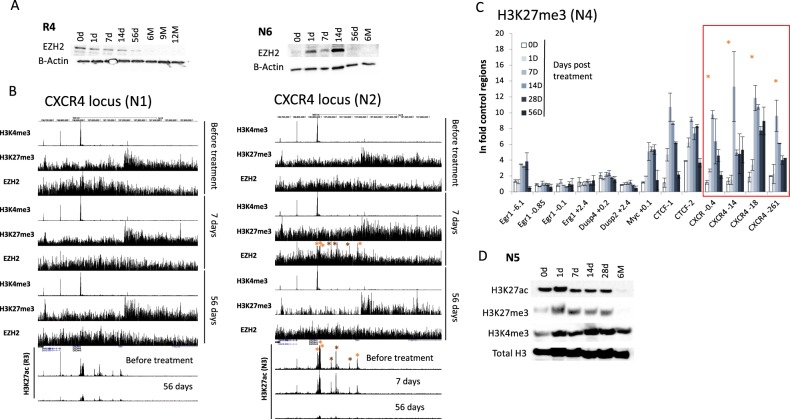
Polycomb-dependent silencing follows Ibrutinib-induced lymphocytosis. **a** Immunoblotting showing the evolution of EZH2 protein level (patients R4 and N6) for up to 1 year on Ibrutinib. **b** Summary of the ChIP-seq data focussing on the CXCR4 locus obtained before treatment and after 7 and 56 days on Ibrutinib. ChIP-seq has been performed for H3K4me3, H3K27me3 and EZH2 (patients N1 and N2) and H3K27ac (patients R3 and N3). **c** Time course of the evolution of the H3K27me3 PTM in selected cis-elements for the first 56 days on Ibrutinib (N4). **d** Immunoblotting showing the evolution of H3K27ac, H3K27me3 and H3K4me3 marks for up to 6 months on Ibrutinib (N5)

EZH2 is a marker of proliferation transiently reactivated in proliferation centre in normal B cells^16,18,19^. Therefore, we hypothesised that EZH2 protein level could serve as a suitable marker of lymphocytosis. EZH2 expression was not correlated with the evolution of B cell count in the PB, but either transiently increased in CLL after 14–28 days on Ibrutinib or present at a moderate level before treatment and going progressively down with prolonged exposure to the drug (Fig. 2a). Then, ChIP-seq experiments were performed for EZH2, H3K4me3 and H3K27me3 at 0, 7 and 56 days after the first treatment administration for N1 and N2 patients and H3K27ac for patients N3 and R3 (Fig. 2, Supplementary Fig. S1). Within H3K27me3-low regions, H3K27ac and/or H3K4me3 peaks corresponding to clusters of transcribing genes were observed as seen for the CXCR4 locus and two genomic regions randomly chosen and centred on the FOXP1 and BCL2 genes, respectively (Fig. 2b, Supplementary Fig. S1). In patient N2, in response to Ibrutinib and correlated with lymphocytosis, we observed, at 7 days, a transient and genome-wide increase in EZH2 recruitment and H3K27me3 at “active” regions (Fig. 2b, Supplementary Fig. S1B, D). This transient increase in H3K27me3 was also detected by conventional ChIP in six randomly chosen patients from the trial as shown for patient N4 (Fig. 2c). In contrast, in patient N1, EZH2/H3K27me3 enrichment within “active” regions was seen at the time of treatment initiation (Fig. 2b, Supplementary Fig. S1A and C). For this patient, the enhanced level of H3K27me3 correlating with EZH2 recruitment was subtler than when observed post treatment.

In addition, independently of the initial EZH2 protein level, prolonged and continuous exposure to Ibrutinib-induced loss of EZH2 protein and H3K27me3 (Fig. 2a, d, Supplementary Fig. S2B). Similarly, H3K27ac, which antagonises H3K27me3, disappeared from 56 days after treatment initiation in contrast to the H3K4me3 mark which was relatively stable upon prolonged drug exposure (Fig. 2b, d, Supplementary Fig. S2A). In conclusion, these data show that long-term Ibrutinib treatment induced a severe reduction of both histone H3 lysine 27 acetylation and methylation together with EZH2 protein disappearance in CLL cells.

### In depth analysis of EZH2 recruitment to active cis-regulatory elements

**Fig. 3:**
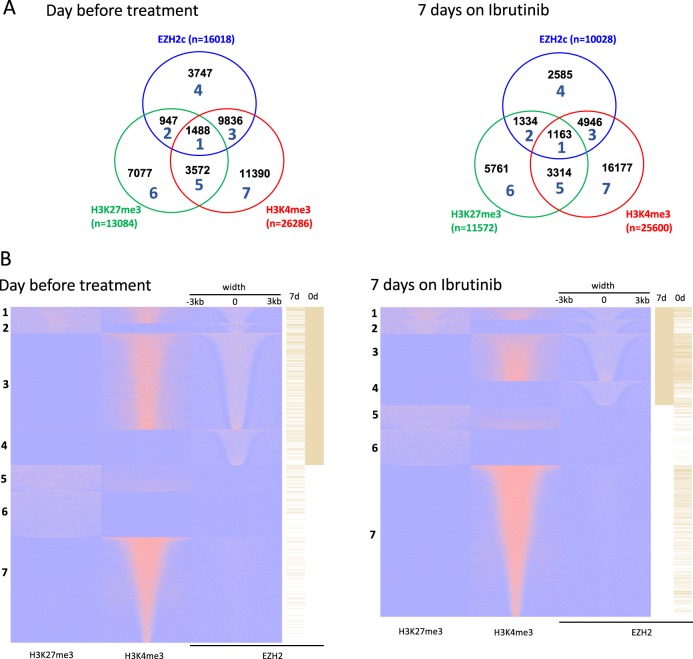
EZH2 redeployment from silenced to open chromatin. **a** Number of cis-regulatory elements associated with EZH2, H3K4me3 and/or H3K27me3 before and after 7 days on Ibrutinib according to the ChIP-seq analysis for N1. **b** Heat map showing the repartition of the seven subsets presented in **a**. The barcodes on the right part of the figure represent EZH2 ChIP-seq data at 7 and 0 days

To study EZH2 recruitment and H3K27me3 deposition in “active” chromatin domain in more detail, we separated the cis-regulatory elements positive for EZH2, H3K4me3 and/or H3K27me3 into seven subgroups before treatment and after 7 days on Ibrutinib. This analysis was performed with the data obtained from patient N1 (Fig. 3a, b) as the remarkable genome-wide increase of H3K27me3 in patient N2 at 7 days was preventing an accurate quantification of positive peaks vs. background.

When EZH2 was targeting regions enriched for H3K27me3, we observed only 2.5% increase in EZH2 bound cis-elements between 0 and 7 days (subgroups 1 and 2) (Fig. 3a, b). In contrast, if EZH2-targeted regions were negative for H3K27me3, EZH2 occupancy decreased by 38% (EZH2-only, subgroup 4) and 50% (EZH2 and H3K4me3, subgroup 3) after 7 days on Ibrutinib. An opposite evolution (30% increase) was observed for H3K4me3-only regions (subgroup 7) (Fig. 3a, b). Moreover, EZH2 was redeployed from H3K4me3 high promoters to H3K27me3-enriched region as highlighted with EZH2 barcodes (subgroups 2 and 4) (Fig. 3b). Altogether, these data indicated that the mechanism of EZH2 dissociation from active clusters described above was partially due to a redeployment of EZH2 to repressed chromatin in addition to a global decrease in EZH2 protein associated with chromatin.

Next, we analysed the intersection between our peak libraries and merged transcription factors ChIP-seq data obtained from a publicly available database (ReMap). This analysis, referred to as ReMap and Epigenetic Marks Intersection (REMI), calculates the number of cis-regulatory elements enriched for a specific histone mark and containing a validated consensus sequence corresponding to a specific transcription factor. It allowed us to generate “signatures” associated with determined peak populations and to assess the relative variation of these signatures when comparing one peak population to another (Supplementary Figs. S3 and 4). For example, we established that non-promoter elements were enriched for transcription factors expressed in B cells and important for B cell function including SMAD1, IKZF1, BATF or BCL11A^22–25^, and promoter elements enriched for proteins like KDM5A/B, IRF3, TAF3 and GTF2B (Supplementary Figs. S3 and 4).

Using REMI analysis, we observed that before treatment, H3K4me3^+^/EZH2^+^ cis-elements were enriched for factors detected at promoters and depleted from specific categories of peaks enriched for SMAD2/3/4, SOX2 and EZH2/SUZ12 (Supplementary Fig. S5A–D). It was at first surprising to find EZH2-bound regions depleted from the EZH2/SUZ12 validated sequences assembled in the ReMap database. However, when analysing the repartition of EZH2-assembled sequences from ReMap compared to the seven subgroups identified in Fig. 3, we determined that these sequences were mainly associated with bivalent promoters (Supplementary Fig. S5B). Moreover, these sequences were also the most represented at CpG islands when compared to promoter regions (Supplementary Fig. S6B). We concluded that our ChIP-seq experiments were showing an under-representation of EZH2 in regions where chromatin was “permanently” repressed (H3K27me3 was stable with time on Ibrutinib). These regions are enriched for the EZH2-validated sequences from ReMap (e.g. ChIP-seq performed on stem cells or partially dedifferentiated cell lines).

SMAD2, 3 and 4 were also found depleted from the H3K4me3^+^/EZH2^+^ fraction. Most of SMADs downstream genes were associated with signal transduction and response to stimuli and included FOXO1 and FOXO3, two transcription factors that mediate quiescence^26,27^ (Supplementary Figs. S5A, D, E, S6A, Supplementary Table 2.1). Moreover, only two out of six samples of CLL cells showed a significant CXCR4 and RAC1 increased mRNA level in response to both TGFß (SMAD-activating signal) and WNT-signalling activation in vitro (Supplementary Fig. S5F) as described from previous reports^28^. These data suggest that CLL cells might be sensitive to TGFß and WNT signalling during very specific window(s) of their life cycle. In conclusion, our data suggest that some H3K4me3^+^-regulatory elements might be actively protected from this observed genome-wide PRC2 recruitment.

### Coordinated changes in H3K4me3 peak repartition and intensity at selected cis-elements in Ibrutinib-treated CLL cells

**Fig. 4:**
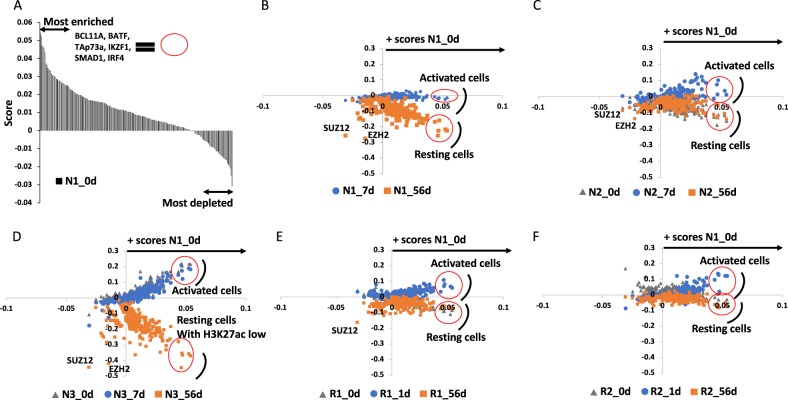
Ibrutinib-dependent and Ibrutinib-independent repartition of H3K4me3 peaks. **a** Cis-elements enriched for the H3K4me3 mark were screened for the presence of validated binding sites for transcription factors and co-regulators (ReMap database). For each protein of the database, data were expressed as a score calculated using the highlighted equation allowing comparison between N1 and an average of N1–3, R1–2 samples before treatment. Scatter diagrams representing a comparison between REMI’s scores for samples (**b**) N1, (**c**) N2, (**d**) N3, (**e**) R1 and (**f**) R2 and reference profile N1 at 0 day, with time points before treatment in grey triangles, at 56 days in orange rectangles and at 1 or 7 days in blue circles. Details of the calculated scores are presented in Supplementary Table 5

To assess the evolution of the repartition of the H3K4me3^+^ peaks between patients and in reponse to Ibrutinib, we generated scores for each set of data compared to the average scores obtained for the five samples analysed before treatment and used patient N1 before treatment as a reference profile as described (Supplementary Fig. S7, Supplementary Table 5). In this reference sample, EZH2 recruitment to active cis-elements correlated with increased scores for transcription factors enriched at non-promoter elements and slightly decreased ones for factors like EZH2 and SUZ12 compared to the average scores (Fig. 4a, Supplementary Table 5). Interestingly, comparable profiles were observed for CLL cells from patients treated for a week or less with Ibrutinib (blue circles) for which the scores for transcription factors enriched at non-promoter elements are similar or even higher (Fig. 4b–f). In contrast, after 2 months treatment (orange squares), the number of H3K4me3 peaks at non-promoter and EZH2/SUZ12-associated cis-elements were significantly reduced (Fig. 4b–f). These differences were more accentuated in TN compared to RR patients (Fig. 4f), the latter category having a prolonged phase of lymphocytosis compared to the former (Fig. 1b).

Furthermore, we identified 253 genes closest to H3K4me3 peaks found in all five blue profiles but in none of the orange ones and determined that they were coding for proteins mainly involved in cell proliferation, response to external stimulus and metabolic process (Supplementary Table 5) suggesting that these peaks were charactersitic of activated cells coming from the proliferation centre of the lymph node (Fig. 4b–f). Interestingly, this family of peaks could be found before treatment in N1 and N3 but not in N2, R1 and R2 (Fig. 4b–f) suggesting episodes of lymphocytosis prior treatment.

**Fig. 5:**
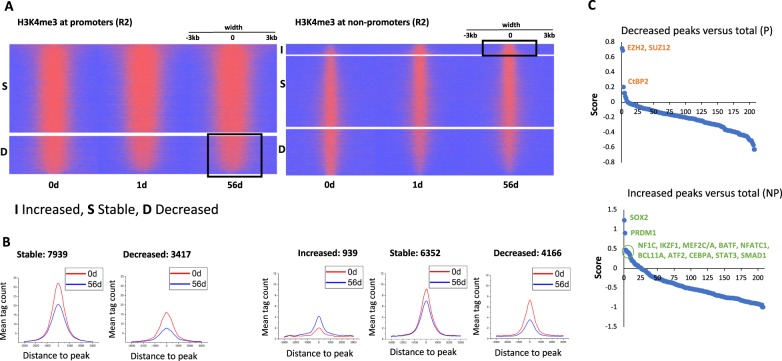
H3K4me3 peak intensity changes in response to Ibrutinib. **a** Heat map of the H3K4me3 peaks in promoters and non-promoters after 0, 1 and 56 days on Ibrutinib (patient R2). Peaks are ranked based on fold change (FC) at 56 days vs. 0 day and separated based on FC = 1.5 (largest to smallest). **b** Corresponding average peak intensities are shown for the stable, decreased and increased peak fractions. **c** REMI analysis-derived score for the factors associated with the decreased promoters (left) and increased non-promoter (right) fractions at 56 days (Eq. (2) in supplementary methods). Data are ranked from the most enriched to the most depleted

Having analysed H3K4me3 peak repartition evolution, we then focussed on the evolution of their intensity with time on Ibrutinib. H3K4me3 global intensity stayed stable with time during treatment (Fig. 2d), but taken individually, H3K4me3 peak intensity showed variation at 1 and 56 days on Ibrutinib compared to before treatment (Fig. 5a, b). To determine if these variations were purely stochastic or were co-ordinately affecting specific families of peaks, we compared the composition of the bottom (most decreased compared with before treatment) and top (most increased compared with before treatment) peaks at 56 days for the promoter-associated and non-promoter-associated H3K4me3 marks, respectively (black boxes), with the one obtained for the total amount of H3K4me3 peaks (Fig. 5c). In agreement with changes observed for the repartition of H3K4me3 peaks, the “top” subset was enriched for transcription factors associated with activated B cells and B cell functions (Fig. 5a–c, Supplementary Table 2.2), and the “bottom” subset enriched for promoter regions associated with EZH2/SUZ12, which were identified in patient N1 at bivalent promoters and CpG islands (Fig. 5a–c, Supplementary Table 2.3). CtBP2, which was enriched in the “bottom” subset is not expressed in CLL but has been shown to participate in PRC2 recruitment at active embryonic stem cells during exit from pluripotency^29^, which reinforces the view that EZH2/SUZ12 ReMap libraries were biased toward earlier differentiation stages.

**Fig. 6:**
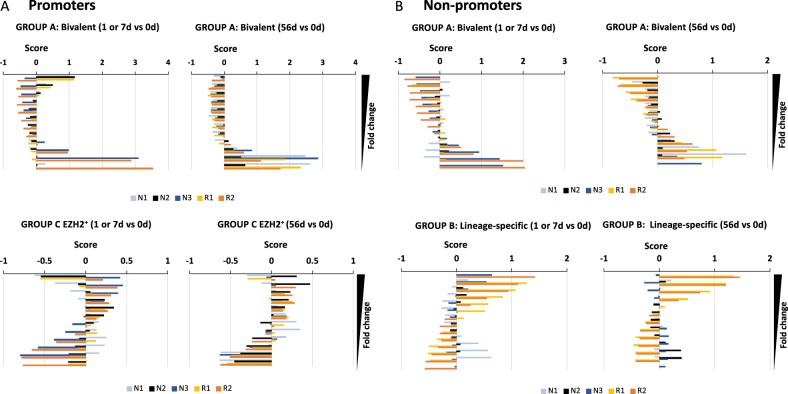
Subclasses of H3K4me3-positive cis-elements are differentially affected by Ibrutinib treatment. (**a**) Promoters and (**b**) non-promoters repartition of the H3K4me3 peaks into the three subclasses, group A (EZH2, SUZ12 and CtBP2), group B (BCL11A, IKZF1, BATF and SMAD1) and group C (KDM5A, IRF3, GATAD1, SNAPC1). The peaks were ranked based on the fold change in magnitude between the time points and binned into subsets containing 1000 peaks. Each of these subsets was analysed in REMI to assess the evolution of the number of peaks associated with the factors from each group **a**, **b**, **c** calculated as score (Eq. (3) in supplementary methods)

We analysed these variations in H3K4me3 peak intensity for patients N1–3 and R1–2 over time (Figs. 5 and 6, Supplementary Fig. S8 and 9). First, the heatmap profiles suggest an important heterogeneity in the amount and intensity of peak changes in response to treatment (Fig. 5a, Supplementary Fig. S8). However, when the ranked peaks (based on fold change) were binned into 1000 peak subsets and the composition of each subset analysed in REMI compared to the composition observed for all the peaks together, we identified four categories of cis-elements behaving co-ordinately. Groups A and B represent the family of peaks enriched in the “bottom” subset (EZH2/SUZ12/CtBP2-containing sequences, bivalent cis-elements) or in the “top” subset (IKZF1, SMAD1, BATF and BCL11A-containing sequences, lineage-specific) for patient R2, respectively (Fig. 5c, Supplementary Fig. S4). Group C (GATAD1, KDM5A, IRF3 and SNAPC1) and D (SMAD2–4) contains transcriptions factors enriched and depleted in the H3K4me3+/EZH2+ subset, respectively (Supplementary Fig. S5A). The peaks from group D were evenly distributed suggesting that these regions were not sensitive to Ibrutinib treatment (Supplementary Fig. S9A). In contrast, group A was found correlated with peaks for which H3K4me3 enrichment was decreasing in promoter and non-promoter regions after 2 months on Ibrutinib (Fig. 6a, b). These regions were mainly bivalent regions (Supplementary Fig. S9B). This effect was observed as early as 1 day for patient R2 or 7 days for patient N3 suggesting variable dynamics in response to treatment but with a common outcome (Fig. 6a, b). Group C was globally depleted from the regions enriched for group A suggesting that these peaks were protected from demethylation indicating that EZH2-recruitment was not followed by epigenetic silencing (Fig. 6a). In group B, patients N1 and N2 showed opposite distribution at 7 days (Fig. 6b) as an indication that H3K4me3 intensity was increasing at enhancer elements targeted by EZH2 (Fig. 2b, Supplementary Fig. S1). For this group, the peak repartition was the same for patients N2, N3, R1 and R2 at early time points suggesting that EZH2-transient recruitment occurred in response to Ibrutinib in four out of the five studied CLL patients (Fig. 6b). Moreover, this enrichment for “lineage specific” peaks in H3K4me3-increased fraction was maintained at 56 days in both RR patients but not in TNs suggesting a difference in the kinetics of Ibrutinib response between these two categories of patients as indicated by the evolution of CLL cell count and H3K4me3 peak repartition (Figs. 1a and 4).

### RR CLL cells may have undertaken partial reprogramming in response to FCR

**Fig. 7:**
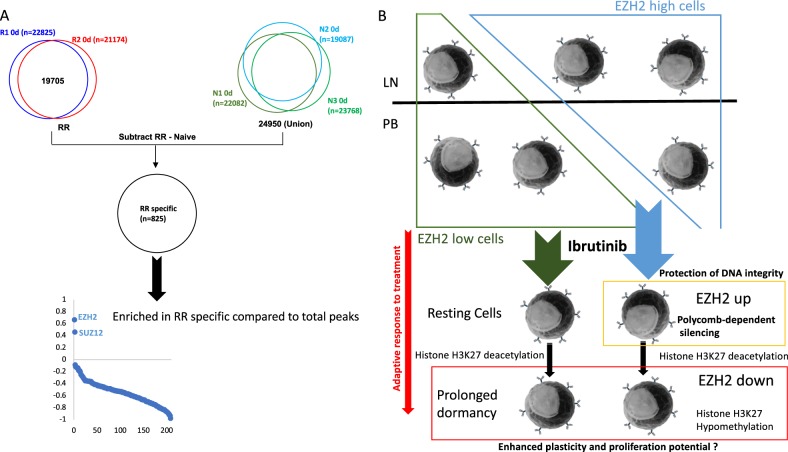
The initial number of bivalent promoters is higher in relapsed/refractory compared to treatment naïve patients. **a** Methodology to isolate H3K4me3 peaks found in both R1 and R2 but not in either of N1, N2 or N3 and REMI analysis of R1 and R2 only peaks shown as score (Eq. (2) in supplementary methods). **b** Summary of the chromatin alterations induced by Ibrutinib. In response to Ibrutinib, proliferation is blocked and two populations of cells are seen depending on the level of EZH2 protein before treatment. Polycomb-dependent silencing is associated with a transient increase in EZH2/H3K27me3 at “active” cis-elements. The H3K27ac and H3K27me3 marks are lost in correlation with the observed global changes in gene expression and a possible increased plasticity and enhanced proliferation potential

To further investigate the differences between the two RR and three TN patients, we selected the 825 RR-specific peaks (Fig. 7a). EZH2 and SUZ12 were the only two factors enriched in this subset suggesting activation of cis-regulatory elements silenced in CLL cells from TN patients (Fig. 7a, Supplementary Fig. S10A-B). Accordingly, these polycomb-associated regions were enriched for genes involved in cell signalling and system development including functions associated with other lineages usually silenced in B cells (Supplementary Fig. S11, Supplementary Table 2.4). This analysis, restricted to “presence or absence” of H3K4me3 peaks, underestimated the differences between RR and TN patients. This is illustrated by the TIAM1 gene locus, where at the promoter H3K4me3 is low but not completely absent for N1, N2 and N3 (Supplementary Fig. S12). Interestingly, these epigenetic differences were not seen at TIAM1 mRNA level which was low in both RR and TN patients before treatment (data not shown).

## Discussion

In CLL, clonal evolution leading to resistance to Ibrutinib has been described^9^, but the lymphocyte count stabilisation after long-term exposure to the drug observed in the majority of Ibrutinib-treated patients cannot be explained solely by the presence of resistant clones^30,31^. This absence of remission correlates with the epigenetic changes we observed, suggesting that cancer cells can also change phenotypically in response to treatment (Fig. 7b). Ibrutinib treatment includes loss of both H3K27ac and H3K27me3 marks in every patient tested. Hypomethylation is a characteristic of quiescent cells and an indicator of enhanced plasticity^32–34^. These changes could participate in the mechanisms of resistance as described previously^35^.

Analysing the dynamics of lymphocytosis following Ibrutinib treatment in CLL cells reveals that the response to Ibrutinib tends to be quicker in TN patients and delayed in RR patients^36^. In this study, this delay can be linked with a significant number of promoters showing bivalency in RR patients but fully silent in TN patients, including TIAM1, which has been shown to mediate chemoresistance to fludarabine in CLL^37^. Maintenance of H3K4me3 at PRC2 targets in RR patients argues for a mechanism of partial reprogramming that takes place during the process of relapse induced by FCR and for which these patients have kept an epigenetic memory which could decrease their sensitivity to Ibrutinib. Further investigation with more CLL samples will be necessary to confirm this point.

EZH2 expression is restricted to proliferative cells in cancer and non-transformed human cells^38^ and, accordingly, to the proliferation centre in B cells^18,19^. Therefore, transient increases in protein level as well as an enhanced interaction with chromatin, and more specifically with H3K4me3^+^ cis-elements in correlation with treatment initiation, can be interpreted by an enrichment of CLL cells accumulating in the blood from the lymph node. Interestingly, a significant level of EZH2 protein and EZH2–chromatin association on the day of treatment initiation could also be seen as an indication of a drug-independent mechanism of lymphocytosis commonly seen in CLL. When EZH2 was measured at day 0, treatment initiation was not provoking any surge in EZH2-expressing cells to the PB suggesting temporary exhaustion of these cells in the lymph node post lymphocytosis. Moreover, H3K27me3 increase at EZH2-bound cis-elements was relatively subtle when EZH2 was associated with chromatin at the beginning of the treatment suggesting that EZH2 might be recruited in an inactivated form. Transcription can repress PRC2 activity through its interaction with nascent transcripts^39,40^ and therefore antagonising PRC2 binding to chromatin^41^. Our data suggest a possible two-step mechanism in which PRC2 is recruited to active cis-elements preventing promoter–enhancer interaction. At this stage, a low level of transcription might prevent PRC2 interaction with chromatin and H3K27 acetylation/methylation switch. Then in response to Ibrutinib, additional repression mechanisms may play a role in favour of a strong repressive state and H3K27me3 deposition. A similar mechanism has been described in Drosophila in response to heat shock accompanied by a global chromatin 3D reorganisation driven by polycomb^42^.

In addition, an in-depth analysis of the evolution of H3K4me3 peak repartition and intensity during the first 2 months of treatment revealed epigenomic plasticity both Ibrutinib-dependent and Ibrutinib-independent. First, regions targeted by EZH2 were protected from H3K4me3 demethylation and, for non-promoter elements targeted by B-cell-specific transcription factors, correlated with a transient increase in H3K4me3. This may be due to an increase in enhancer-specific noncoding RNA (eRNA) transcription. Second, stable bivalent cis-elements appeared to be among the regions excluded from this transient EZH2 recruitment, which was in agreement with a role for EZH2 in de novo H3K27 methylation and EZH1 for the maintenance of this mark^43^. Moreover, these bivalent cis-elements showed a trend toward reduction of H3K4me3 peak intensity in all patients, suggesting a correlation between a prolonged exit from the cell cycle as a consequence of Ibrutinib treatment and demethylation of both H3K27me3 and H3K4me3 at silenced chromatin.

Some H3K4me3+ cis-elements were specifically protected from EZH2 recruitment and in particular regions potentially targeted by SMAD2–4. TGFß has been shown to be inhibitory at almost any B-cell stage^44^ and may participate in the reorganisation of the transcriptome during the transition from the proliferative to the resting phase. This hypothesis is reinforced by the presence of FOXO1 and FOXO3 in the list of genes protected from EZH2 recruitment and potentially targeted by SMAD2–4. FOXO1 and FOXO3 control a transcriptional programme repressing cell proliferation and promoting resistance to stress and survival^45^. Moreover, the expression of these two transcription factors is downregulated by the BCR signalling^46^. Interestingly, SMAD6 and SMAD7, the two inhibitors of TGFß signalling^47,48^ are also among the genes identified within the EZH2^−^/SMAD2–4^+^ fraction, arguing for a short window of activation of this signal as suggested by the observed variability in CLL response to TGFß1 in vitro. In addition, TGFß-induced quiescence has been recently shown to mediate chemoresistance in squamous cell carcinoma^49^. Altogether, these data suggest that the passage from proliferating to quiescence in response to Ibrutinib is controlled by polycomb and potentially TGFß.

In conclusion, chromatin structure changes dynamically in response to Ibrutinib in CLL cells in a two-step mechanism leading to the loss of H3K27 marks and correlated with the stabilisation of CLL cell count. Histone hypomethylation associated with prolonged exit from the cell cycle has been connected to mechanisms of survival and enhanced plasticity and can be seen as an adaptive response to Ibrutinib. Further investigation will be necessary to assess if these changes provoke a reduced sensitivity to treatment and participate in the mechanism of relapse.

## Materials and methods

### Isolation of CLL cells

Peripheral blood was collected from patients with informed written consent (St. James’ University Hospital, approval number 14/YH/0034). Peripheral blood mononuclear cells (PMBCs) were isolated by density gradient centrifugation using Lymphoprep^TM^ (Axis-Shield). CLL cells were further purified using CD19+ MicroBeads (Miltenyi Biotec). Due to the restricted cell number for each collected time points, purified cells were used for immunoblotting, gene expression or chromatin immunoprecipitation as described below.

### Immunoblot analysis

Cells were lysed in a buffer containing 50 mM Tris–HCl (pH 6.8), 1.5% SDS and 1x protease inhibitor cocktail (ThermoFischer Scientific). Protein concentration was determined using Pierce BCA™ Protein Assay (ThermoFischer Scientific). Protein from each cell-lysate was separated by SDS–PAGE, followed by electrotransfer into a nitrocellulose membrane. Proteins of interest were visualised and quantified after primary antibody incubation using Pierce™ ECL Western Blotting Substrate (ThermoFisher Scientific) and ChemiDoc Imaging System (Bio-Rad). Mean band intensity was calculated using Image Lab software. Primary antibodies were as follows: H3K4me3 (Millipore; 04-745), H3K27me3 (Millipore; 07-449), H3K27ac (Millipore; 07-360) and EZH2 (Diagenode; pAb-039-050) were used as primary antibodies; anti-β-actin (Sigma; A1978) and anti-Total H3 (Abcam; ab1791) were used as loading controls.

### Chromatin immunoprecipitation (ChIP) and ChIP-seq

ChIP was performed as previously described^50,51^ using dynabeads protein G (Invitrogen) with 2.4 mg per 10 ml beads with 1 μg anti-H3K4me3 (Millipore; 04-745), anti-H3K27ac (Millipore; 07-360), anti-H3K27me3 (Millipore; 07-449) and anti-EZH2 (Diagenode; pAb-039-050) antibodies. See Supplementary Table 3 for primer sequences.

For sequencing (ChIP-seq), libraries were prepared using NEBNext^®^ Ultra™ II DNA Library Prep Kit (New England Biosciences). See Supplementary Methods for sequencing run details and Supplementary 4 for QC of these libraries.

### Bioinformatics analysis

See Supplementary Methods for further details. Sequence data from this study have been submitted to ArrayExpress (https://www.ebi.ac.uk/arrayexpress/) under accession number E-MTAB-6410.

## Supplementary information

Supplementary figures

Supplementary methods

Supplementary table 1

Supplementary tables 2

Supplementary table 3

Supplementary table 4

Supplemetary table 5
