# Supplementary material for: Ibrutinib induces chromatin reorganisation of chronic lymphocytic leukaemia cells
Source: Oncogenesis. 2019 May 10;8(5):32. doi: 10.1038/s41389-019-0142-2 (PMC6510766; doi:10.1038/s41389-019-0142-2)
Supplement: Supplementary file 1 — Supplementary figures [file 41389_2019_142_MOESM1_ESM.pdf]

**Supplementary figure S1: EZH2 transient recruitment to active cis-regulatory elements.** ChIP-seq data for the following regions chosen as example, Chr3: 68,257-74,244 (A) N1 and (B) N2 and Chr18: 59,908-61,868 (C) N1 and (D) N2 obtained before treatment and after 7 and 56 days on Ibrutinib. ChIP-seq has been performed for H3K4me3, H3K27me3 and EZH2, (A) and (C) H3K27ac patient R3 and (B) and (D) H3K27ac patient N3.

**Supplementary figure S2: Global loss of H3K27ac and H3K27me3 after prolonged exposure to Ibrutinib.** (A) Heat map for H3K27ac positive regions after up to 56 days on Ibrutinib. Peaks are ranked based on fold change (FC) at 56d versus 0d and separated based on FC = 1.5 (decreased at the bottom, increased at the top). Average peak intensities for the corresponding peak fractions are shown on the right part of the figure. (B) Quantification of immunoblot for H3K27me3 (Two RRs and 2 TNs) up to 12 months on Ibrutinib.

**Supplementary figure S3: Promoters and non-promoters are occupied by different families of transcription factors and co-regulators.** Cis-elements enriched for the H3K4me3 mark were screened for the presence of validated binding sites for transcription factors and co-regulators (ReMap database). For each protein of the database, data are expressed in total amount of sequences containing a validated binding site for the specified factor and sorted based on the data obtained for promoters (proteins with the highest number of associated sequences on the left). For example, the transcription factor SPI1 is found in 4322 promoters and 4552 non-promoters cis-elements enriched for H3K4me3 in CLL cells (R2).

**Supplementary figure S4: Non-promoter sequences are enriched for DNA-binding motifs of B-cell specific transcription factors.** Cis-elements enriched for the H3K4me3 mark were screened for the presence of validated binding sites for transcription factors and co-regulators (ReMap database). For each protein of the database, data were expressed as a score calculated using the highlighted equation allowing comparison between promoter and non-promoter sequences.

**Supplementary figure S5: H3K4me3+/EZH2+ sequences are depleted from SMAD2-4-containing and bivalent cis-regulatory elements.** (A) Comparison between peak scores obtained from REMI analysis separating EZH2 positive and negative sequences before treatment in N1 (equation (2) in supplemental methods). Peaks associated with (B) EZH2, (C) KDM5A and IRF3, (D) SMAD4 shown as a ratio to the total number of peaks (REMI analysis) in the 7

subgroups of peaks as analysed for N1. (E) Methodology to isolate H3K4me3-only peaks associated with SMAD proteins in N1 and N2. (F) *In vitro* analysis of CLL cells response to TGFB1 and WNT5A. Cells were treated for 4 hours with the indicated molecules and CXCR4 and RAC1 mRNA levels were measured.

**Supplementary figure S6: Cis-elements containing validated SMAD binding sites are protected from EZH2 recruitment.** (A) Gene ontology analysis of the peaks selected in Supplemental figure 5E. The red and blue squares correspond to the presence or absence of a gene, respectively. (B) REMI analysis comparing score for promoters versus CpG islands (equation (2) in supplemental methods). The bed file for promoters was generated in R with 1000 bp upstream and 500 bp downstream of transcription start site (TSS) for hg19 (UCSC). The CpG island bed file was also downloaded from UCSC (see Material and Methods).

**Supplementary figure S7: Creation of a reference profile for H3K4me3.** Score for N1 versus the average of N1-3, R1-2 patients before treatment. Cis-elements enriched for the H3K4me3 mark were screened for the presence of validated binding sites for transcription factors and co-regulators (ReMap database). For each protein of the database, data were expressed as a score calculated using the highlighted equation. Details of the calculated scores are presented in supplementary table 5.

**Supplementary figure S8: H3K4me3 positive cis-elements are differentially affected by Ibrutinib treatment.** (A) promoters and (B) non-promoters heat maps for R1, N1, N2 and N3. Peaks were ranked based on fold change (FC) at 56d versus 0d and separated based on FC=1.5 (decreased at the bottom, increased at the top).

**Supplementary figure S9: Subclasses of H3K4me3 positive elements are differentially affected by Ibrutinib treatment.** (A) Promoters and non-promoters repartition of the H3K4me3 peaks for SMAD2,3 and 4 (group D). The ranked peaks were binned into 1000 peak subsets based on fold change and each subset analysed in REMI to assess the evolution of the number of peaks associated with the factors from SMAD subclass calculated as score (equation (3) in supplemental methods). (B) Heat maps including H3K4me3 at 0 and 56 days as well as H3K27me3 before treatment. The peaks are ranked based on H3K27me3 intensity. Regions positive for EZH2 (ReMap) are shown as barcodes on the right part of each heat map.

**Supplementary figure S10: Evolution of H3K4me3 peaks number in CLL cells from patients the day before the start of Ibrutinib treatment** (A) Variation in the number of H3K4me3 peaks associated with each factor of the ReMap database between N1-3 and R1-2 before treatment. The data were sorted from the most variable to the least variable. (B) Number of H3K4me3 peaks associated with EZH2 or SUZ12 for N1-3 and R1-2 before treatment.

**Supplementary figure S11: Identification of genes fully silenced in treatment-naïve patients only** Gene ontology analysis for R1 and R2-only specific peaks, as described in figure 6A associated with EZH2 (annotated to the closest gene). The red and blue squares correspond to the presence and absence of a gene, respectively.

**Supplementary figure S12: TIAM1 locus is silenced in treatment naive but not in relapsed/refractory patients before treatment.** ChIP-seq data for H3K4me3 and H3K27me3 in TN and RR patients at the TIAM1 locus.

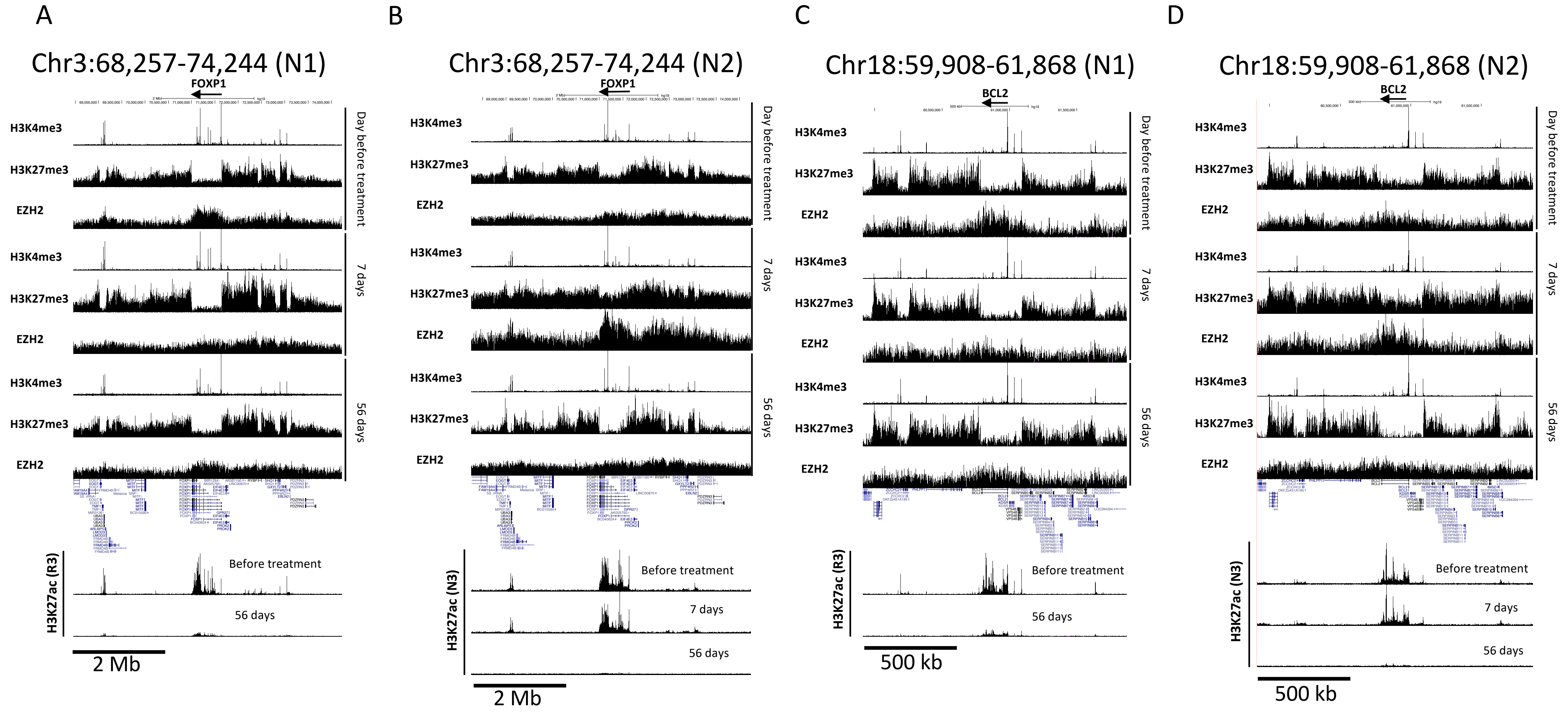


**Supplementary figure S1**

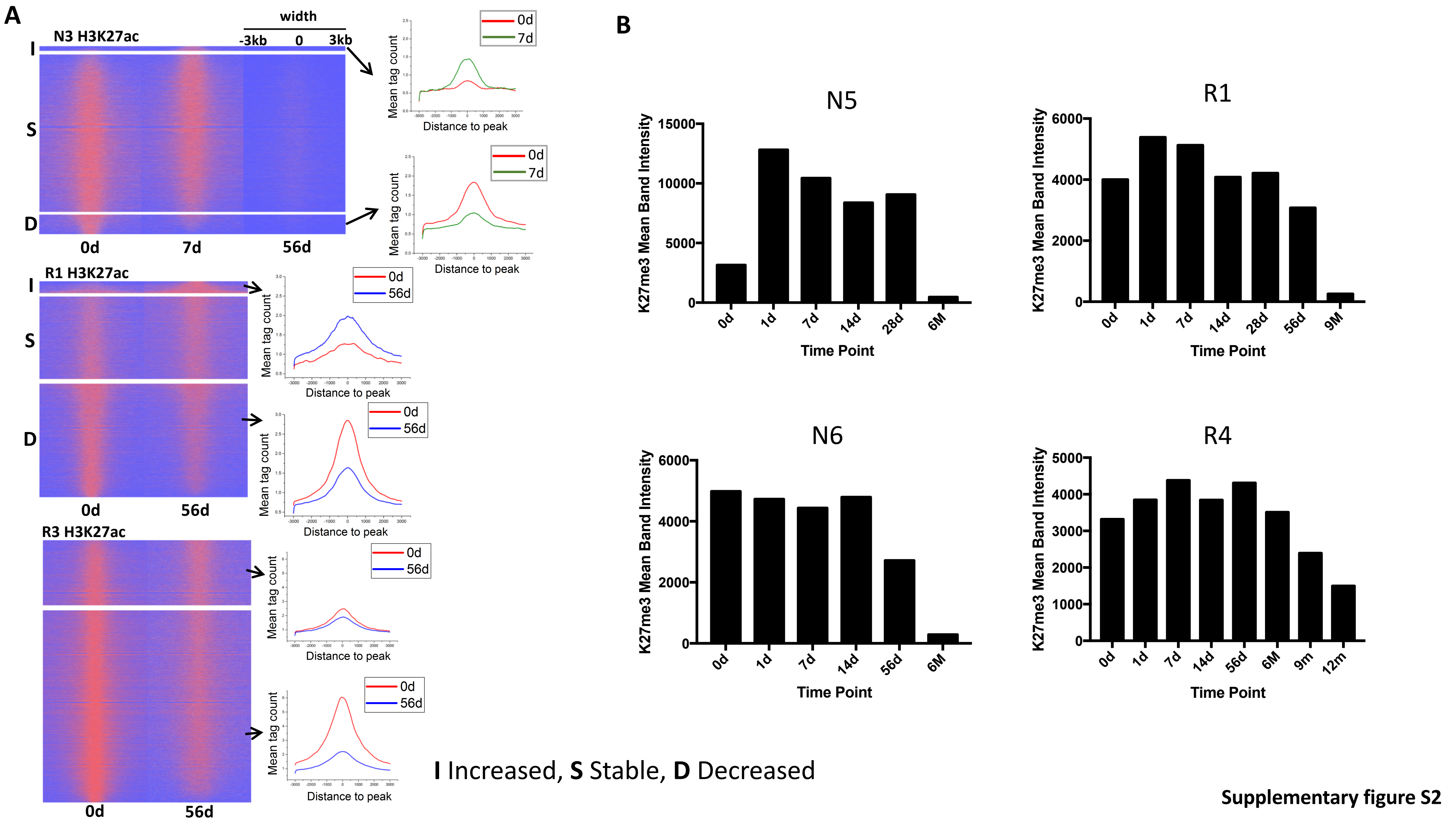


**I** Increased, **S** Stable, **D** Decreased

**Supplementary figure S2**

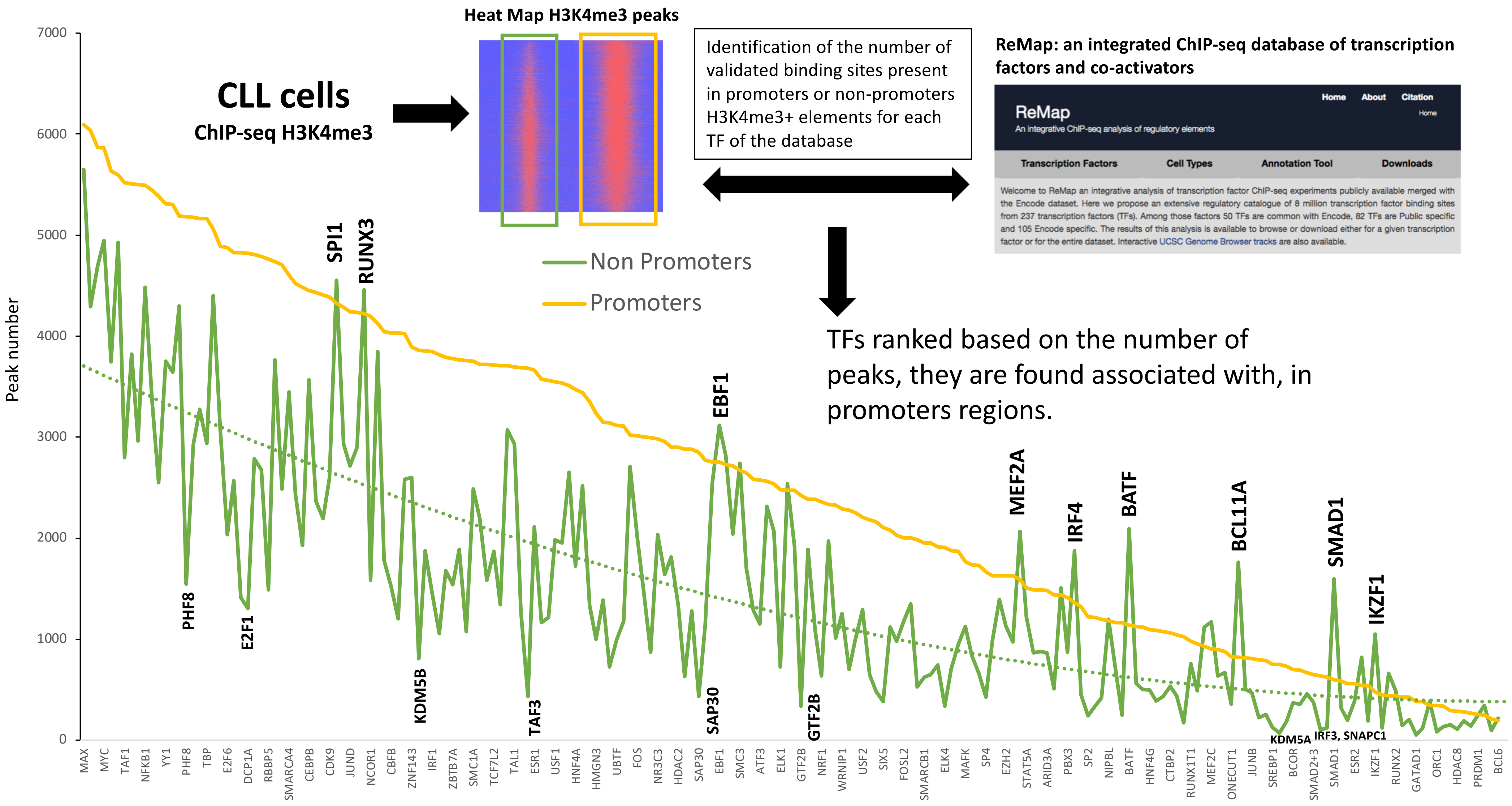


**Supplementary figure S3**

## Calculation of a score to compare the variation in binding-factors composition of H3K4me3+ sequences between non-promoter and promoter regions

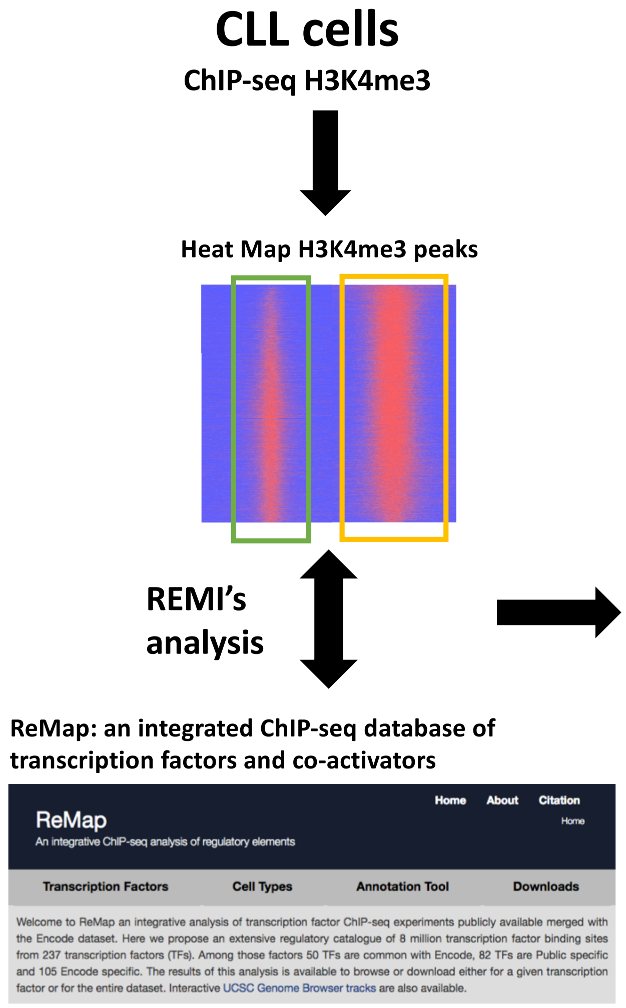


The **SCORE** for a certain TF can be described by the following equation:

$$S_i = \frac{NP_i}{NP_t} / \frac{P_i}{P_t} - 1$$

where $NP_t$ and $P_t$ are the total number of validated-binding sites for the non-promoter and promoter groups, respectively. $NP_i$ and $P_i$ are the number of non-promoter and promoter sequences containing a validated-binding site for the TF $i$, respectively.

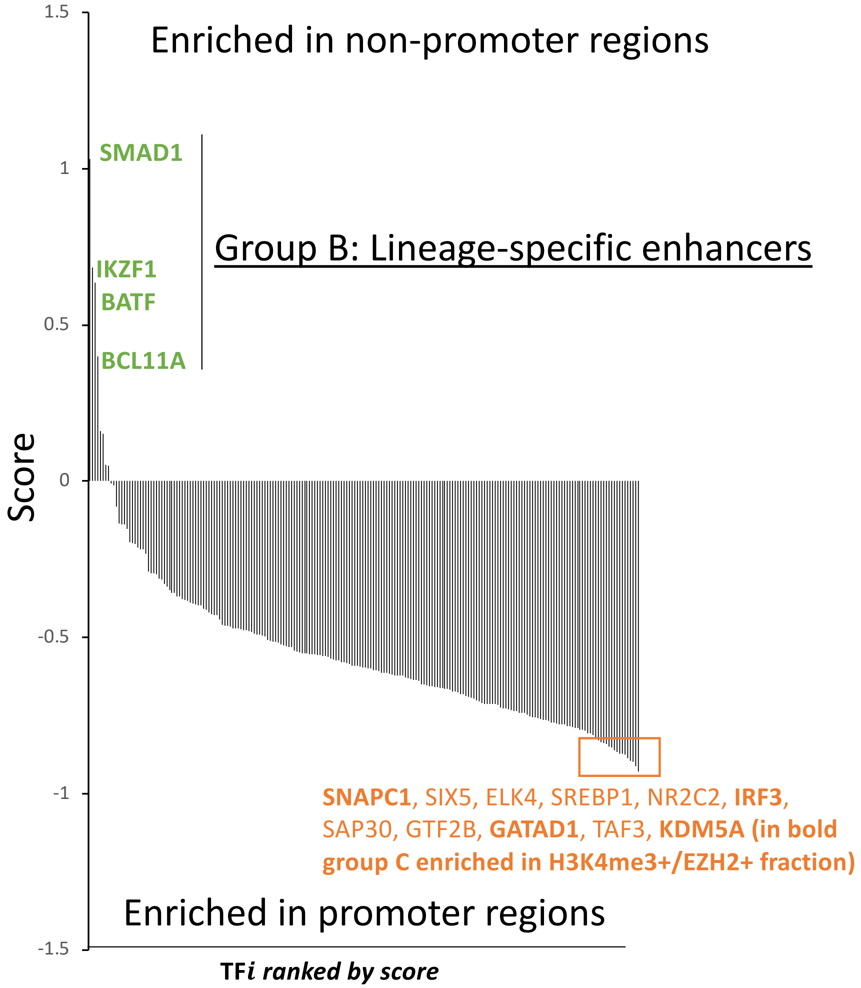


**Supplementary figure S4**

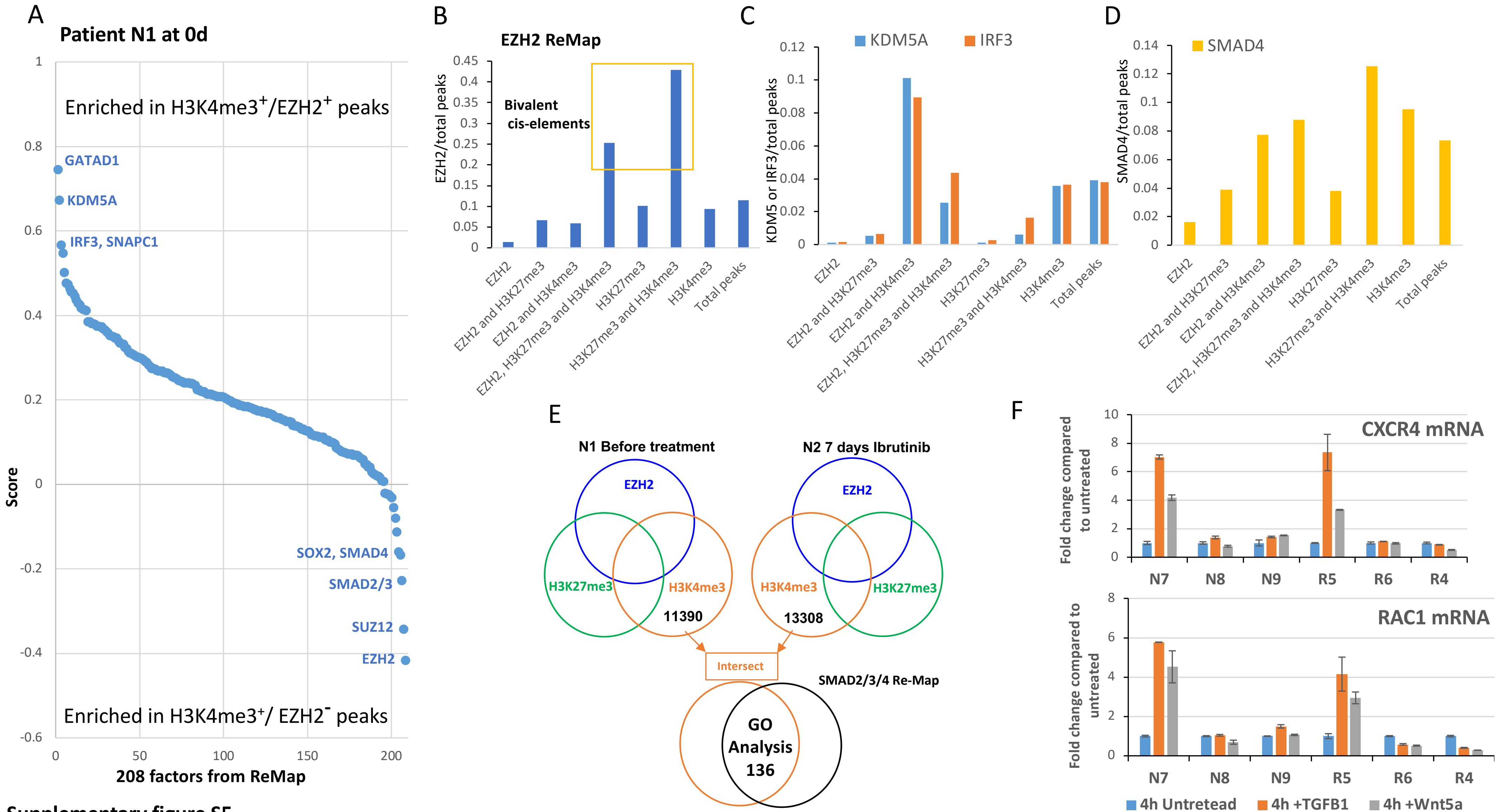


**Supplementary figure S5**

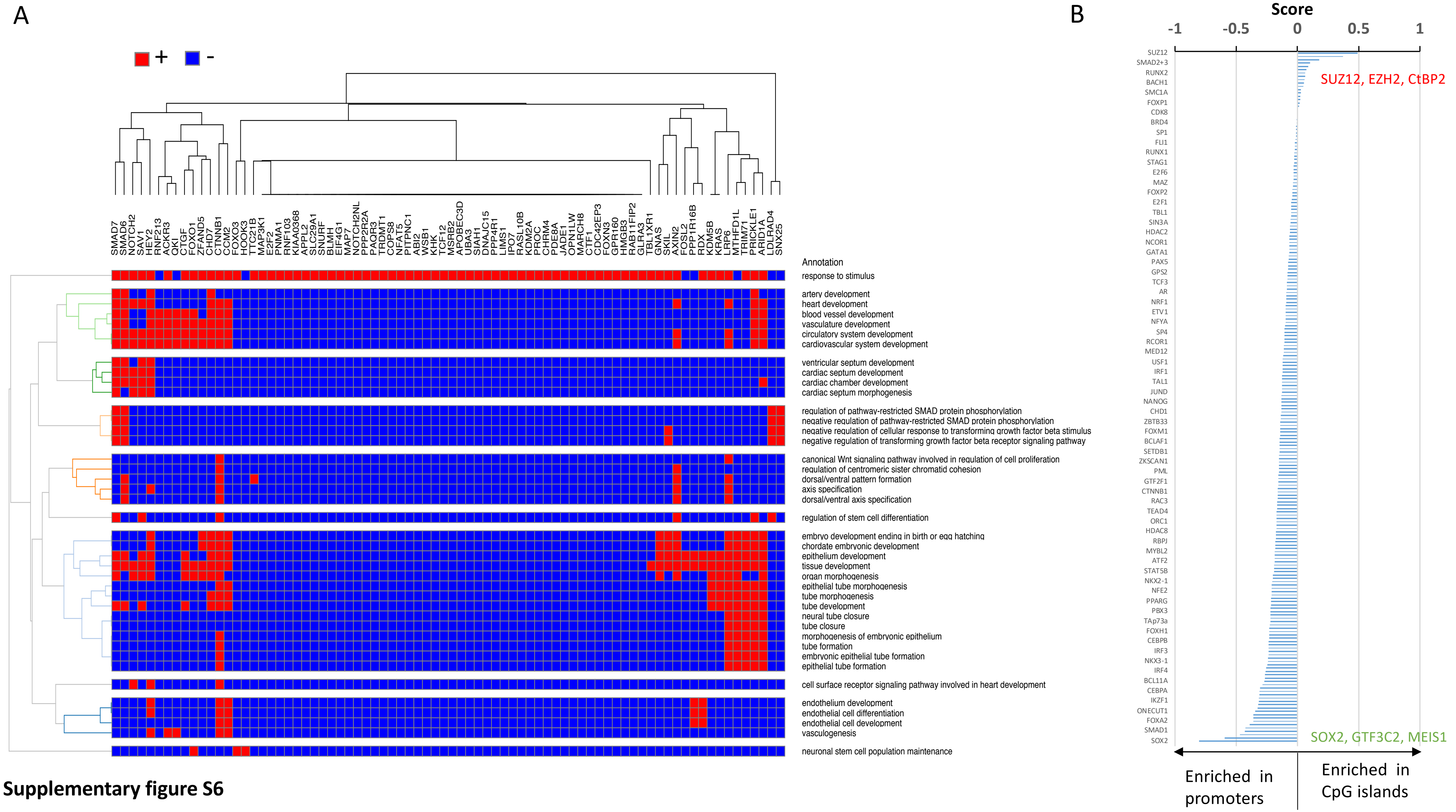


**Supplementary figure S6**

Data from N1 has been chosen because it represents the “middle point” when compared to the other data.

|  | R1 | N2 | R2 | N3 | N1 | Average |
|---|---|---|---|---|---|---|
| ARID3A | 3261 | 3276 | 3392 | 3689 | 3481 | 3419.8 |
| ARNT | 803 | 753 | 861 | 854 | 834 | 821 |
| AR | 11134 | 10900 | 11612 | 11732 | 11502 | 11376 |
| ATF1 | 4099 | 4027 | 4162 | 4328 | 4179 | 4159 |
| ATF2 | 6892 | 6523 | 7114 | 8128 | 7414 | 7214.2 |
| ATF3 | 6573 | 6188 | 6750 | 6727 | 6557 | 6559 |
| ZNF143 | 10747 | 9781 | 11324 | 11045 | 10773 | 10734 |
| ZNF263 | 7566 | 6968 | 7876 | 7454 | 7434 | 7459.6 |
| ZNF76 | 2764 | 2765 | 2969 | 2856 | 2859 | 2842.6 |
| Total peaks | 21174 | 18398 | 22025 | 23623 | 21836 | 21411.2 |

**REMI analysis**

The **SCORE** for a certain TF can be described by the following equation:

$$S_i = \frac{N1_i}{N1_t} / \frac{A0_i}{A0_t} - 1$$

where $N1_t$ and $A0_t$ are the total number of validated-binding sites for the N1 peaks and average peak number for No Ibrutinib controls, respectively. $N1_i$ and $A0_i$ are the number of N1 peaks and average peak number for No Ibrutinib controls containing a validated-binding site for TF$i$, respectively.

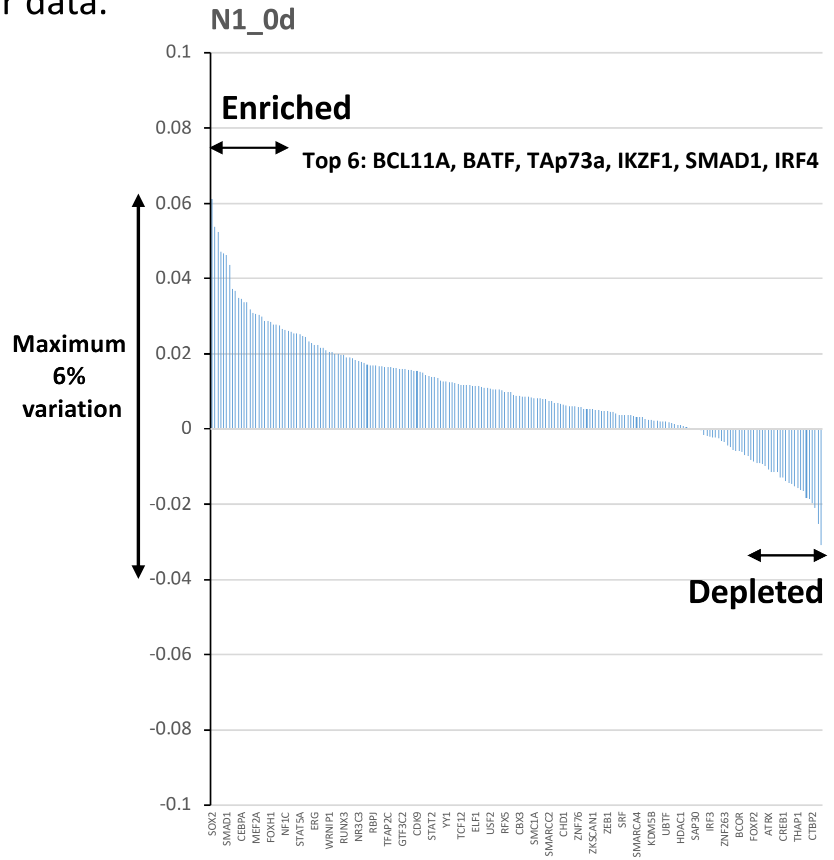


**Supplementary figure S7**

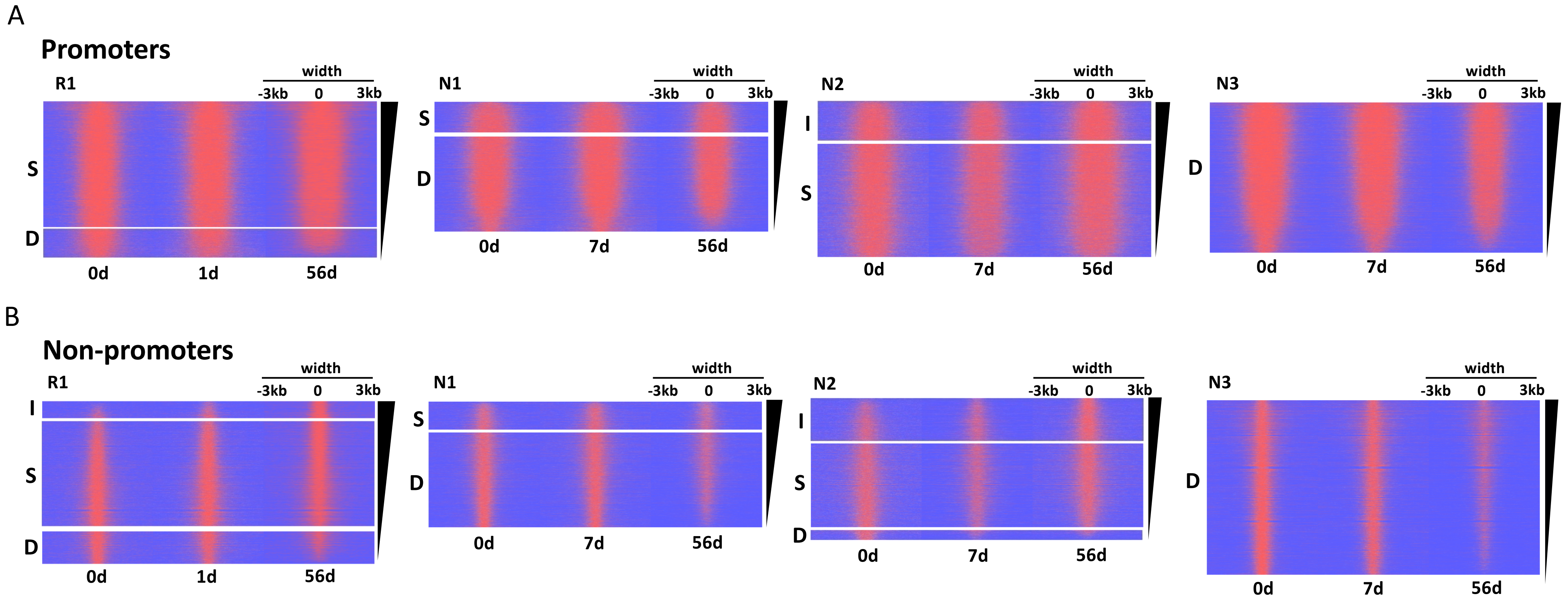


**I** Increased, **S** Stable, **D** Decreased

Supplementary figure S8

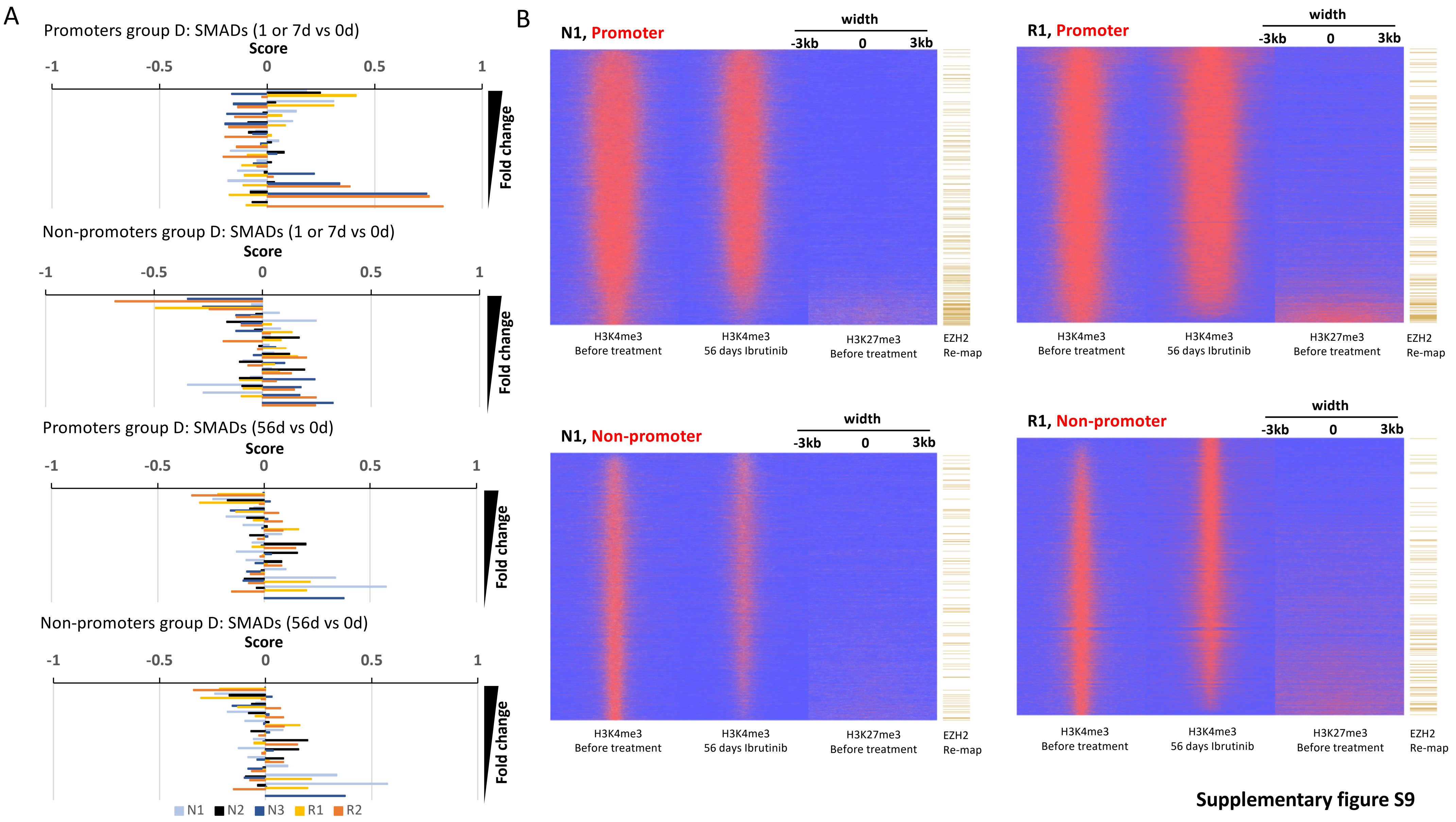


Supplementary figure S9

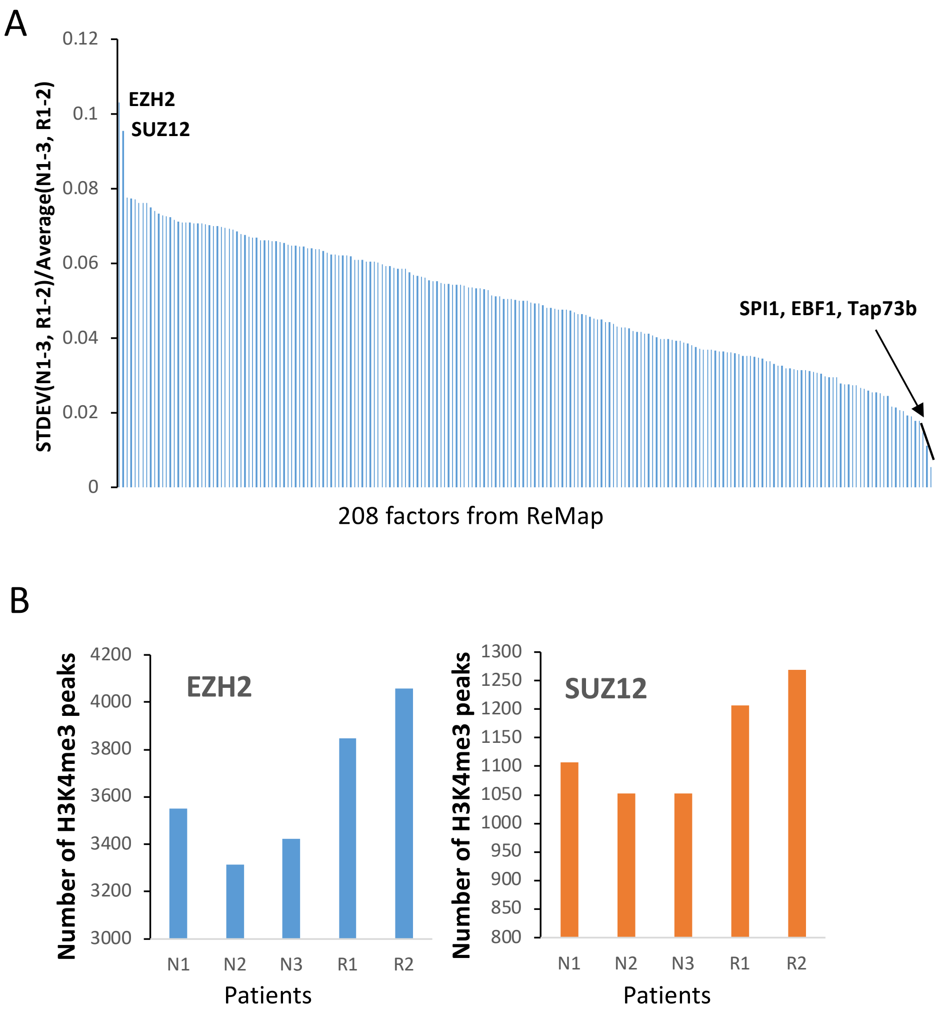


**Supplementary figure S10**

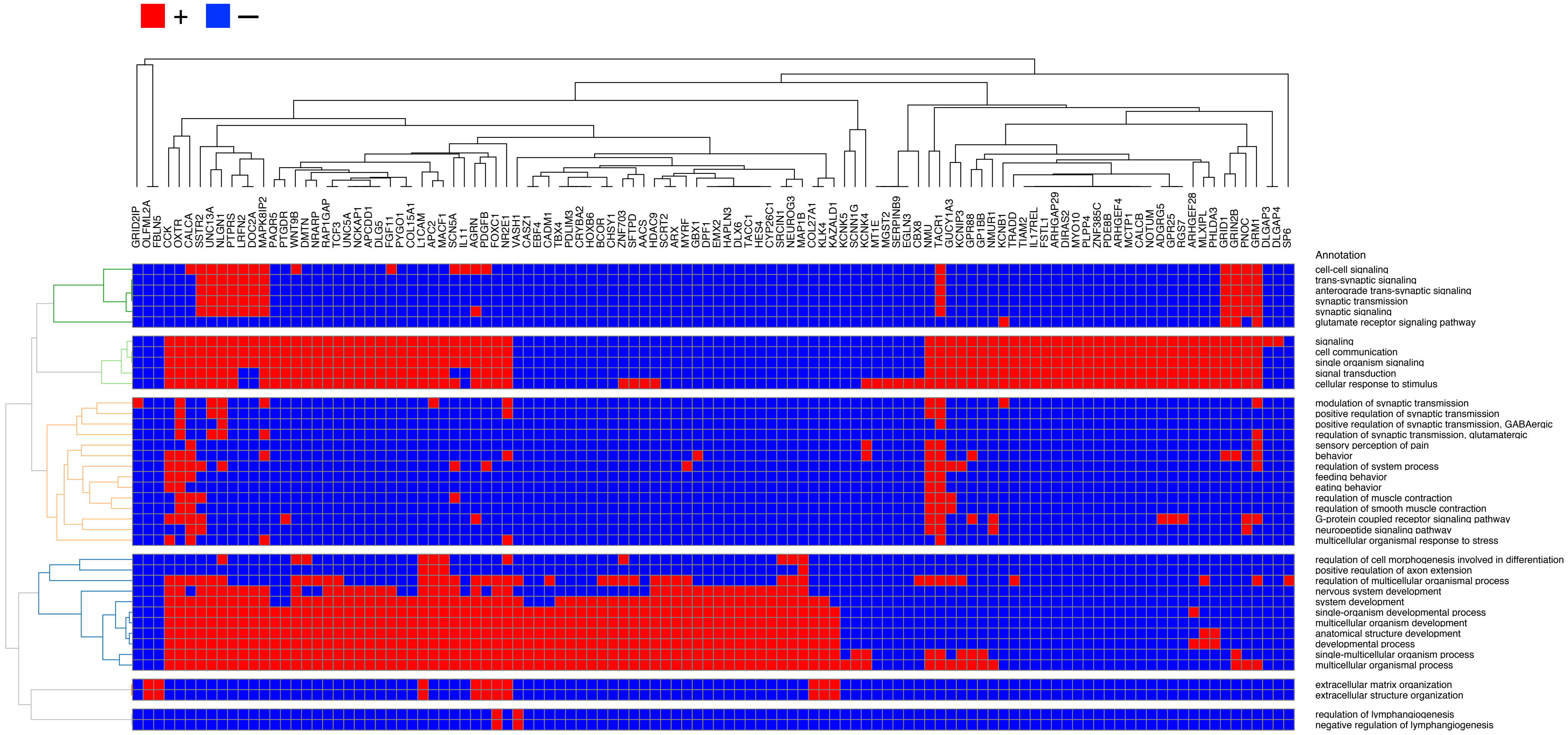


**Supplementary figure S11**

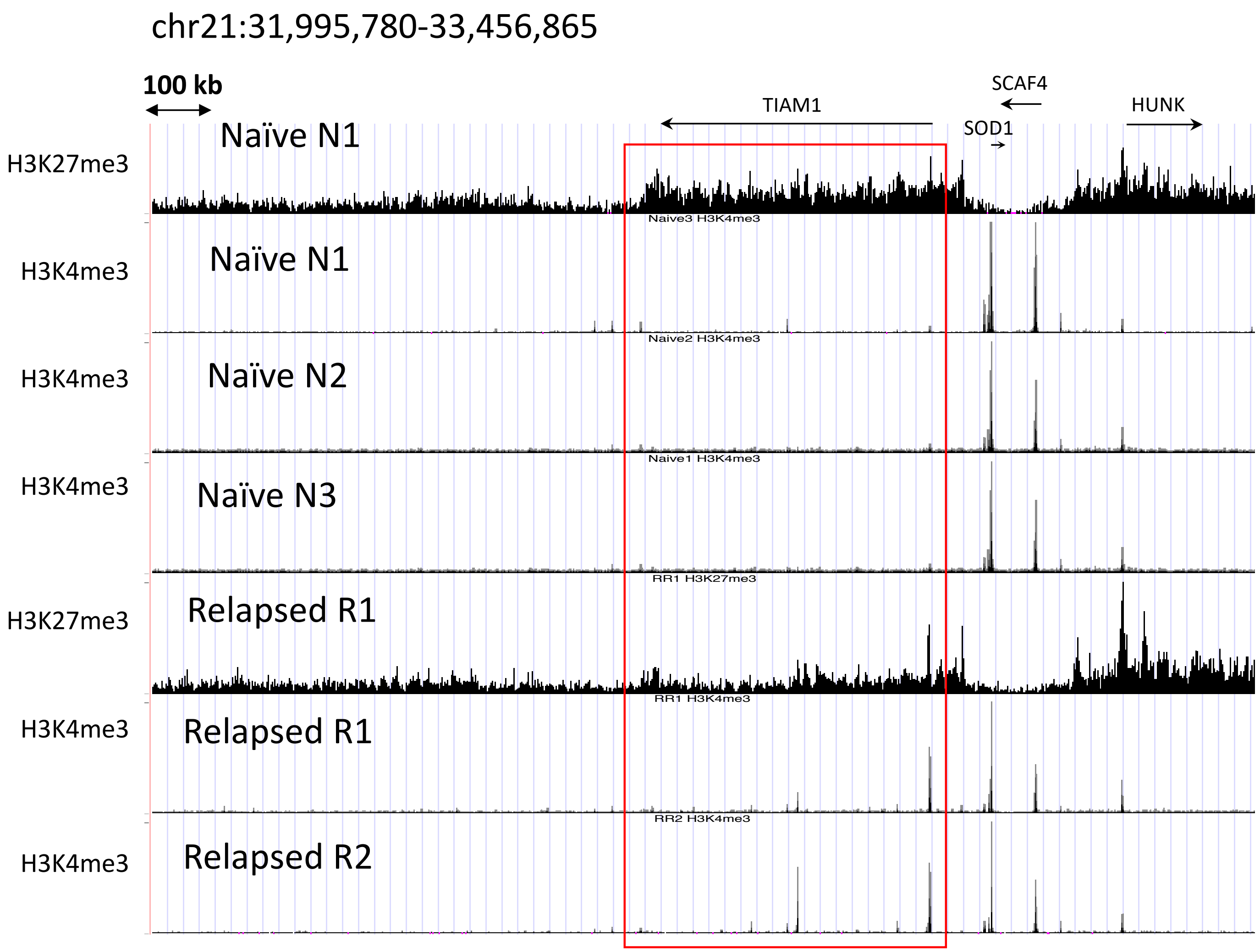


**Supplementary figure S12**
