# Supplementary material for: Ibrutinib induces chromatin reorganisation of chronic lymphocytic leukaemia cells
Source: Oncogenesis. 2019 May 10;8(5):32. doi: 10.1038/s41389-019-0142-2 (PMC6510766; doi:10.1038/s41389-019-0142-2)
Supplement: Supplementary file 2 — Supplementary methods [file 41389_2019_142_MOESM2_ESM.pdf]

Methods

Chromatin Immunoprecitiation and ChIP-seq analysis

For patients N3, R2 and R1 (H3K4me3 1d and H3K27ac), single 50 bp-read sequencing runs were sequenced on an Illumina HiSeq2000 (Duke University Genomics Core Facility). For patients N1, N2 and for patient R1 (H3K4me3 0d, 56d and H3K27me3) single 75bp-read and 50bp-read sequencing runs were performed on an Illumina NextSeq 500 (University of Leeds NGS Facility), respectively.

The analysis of raw CHIP-seq data was performed using human genome hg19 as a reference, single-end (SE) reads with the corresponding input control files. Technical sequences were trimmed by Trimmomatic [1] with the following settings: '6:15:15 TOPHRED64 MAXINFO:51:0.8 MINLEN:10'. The quality before and after the trimming was assessed by FASTqc (https://www.bioinformatics.babraham.ac.uk/projects/fastqc/). Alignment of the trimmed reads to hg19 was done by bowtie2 [2] using the option '--very-sensitive'. Samtools (http://samtools.sourceforge.net/) was used to convert sam files to bam, sorting and indexing as well as filtering the quality by '-q 20'. Peak calling was performed by SICER [3] with the following settings: window=200bp, gap=200bp for H3K4me3 and H3K27ac; window=500, gap=1500 for H3K27me3, FDR = 0.01. The differential peak calling (between the time points) was also performed by SICER using the differential mode with the corresponding settings. The peaks were sorted by the fold change (FC). In order to identify an overlap between the bed files of interest, Bedtools intersect was used [4] with the following options: '-wa -wb and –u'. The default 1bp was used as the minimum required overlap. To identify peaks specific for a certain condition, Bedtools subtract was used with '-A' option.

The bed files were filtered with ATAC-seq in order to identify DNA-accessible regions. The ATAC-seq bed file was generated using the ATAC-seq files [5] that were merged and where all

the duplicate peak positions were removed. ATAC-seq (Assay for Transposase-Accessible Chromatin) identifies regions of nucleosome-free open chromatin using a hyperactive prokaryotic Tn5-transposase. ATAC-seq captures the tissue-specific chromatin activity of regulatory regions, including promoters, enhancers and insulators. In order to identify promoter and non-promoter regions, the bed file for promoter was generated in R with 1000bp upstream and 500bp downstream of transcription start site (TSS) for hg19 downloaded from UCSC, file "TxDb.Hsapiens.UCSC.hg19.knownGene". The file for CpG islands was also downloaded from UCSC.

ReMap and Epigenetic Mark Intersection (REMI)

REMI is a wrapping tool that was developed by us and that allows the analysis of an intersection between the bed files of interest and the transcription factors (TFs) from the publically available online database ReMap [6]. The ReMap bed files for the TFs are available at (*http://pedagogix-tagc.univ-mrs.fr/remap1/*). REMI is written on Python and is based on Bedtools intersect [4]. REMI was used to establish overlapping regions between the bed files of interest and each of the TFs from ReMap. This allowed identifying the enriched and depleted TFs in certain categories of peaks. In order to annotate peaks to the nearby genes, HOMER annotatePeaks.pl was used for hg19 [7].

In order to assess how a certain group of peaks is enriched or depleted for specific transcription factors or co-activators (TF) obtained from the ReMap database, an enrichment score was derived. The score for a certain TF can be described by the following equation:

$$S_i = n_i/u_i - 1, \qquad (1)$$

where i is the number of TF from the ReMap database ($i = 1, \ldots, 208$), n_i and u_i show the enrichment of TF in the selected group of peaks and globally respectively, "-1" stands for separation of the enriched and depleted TFs into positive and negative values respectively.

In the case of individual TF assessment (figure 3C-D, supplemental figure 4B-F, figure 4C-D, supplemental figure 5A, supplemental figure 6B, figure 6B), Equation (1) transforms into:

$$S_i = \frac{N_i}{N_t} / \frac{U_i}{U_t} - 1, \quad (2)$$

where N_t is the number of peaks in the selected group of peaks, N_i is the number of intersections of these peaks with TF i, U_t is the total number of peaks in the union file that also includes the selected group of peaks and U_i is the intersection of the union file with TF i.

In order to identify how each part of the heat maps with ranked peaks is enriched for TFs, the peaks were binned into subsets containing 1000 peaks. The score was derived for each of these binned subsets and for each of the selected groups g of TFs (groups A, B, C, D in Figures 5B, D and supplemental figure 7A). In this case Equation (1) can be written as:

$$S_g^j = \frac{R_g^j}{\sum_{j=1000}^{q} R_g^j} / \frac{\sum_{i=1}^{208} N_i}{\sum_{j=1000}^{q} \sum_{i=1}^{208} N_i^j} - 1, \quad (3)$$

where $j = 1000, \ldots, q$ is the binned subset of peaks (every 1000 out of q), $k = 1, \ldots, m$ is the number of TF in group g that contains m TFs, $R_g^j = \sum_{k=1}^{m} N_k^j$ is the sum of the overlapping peaks number for each TF in the selected group of TFs g, for each binned subset of peaks j.

Heat maps and visualisation

The heat maps for the visual representation of the peaks that changed between the conditions were generated in Gene-E (*https://software.broadinstitute.org/GENE-E/*). For the hierarchical clustering, one minus Pearson correlation was used as a column and row distance metric. The barcode analysis is based on Bedtools [4] and visually demonstrates overlaps between EZH2 peaks obtained from ChIP-seq or ReMap and the peaks sorted by the FC on the heat maps representing peaks intensities. It was used to identify which parts of the ranked list of peaks are enriched or depleted in the peaks corresponding to EZH2. The barcode analysis and

the other downstream analyses such as mean tag counts were done in R and using custom bash scripts. ChIP-seq peaks were visualised in UCSC browser.

Gene ontology analysis

The Gene Ontology (GO) analysis was performed in Cytoscape [8] using BINGO plugin [9]. For the GO analysis the hypergeometric test was performed in order to identify enriched biological processes. A significance level for the Benjamini and Hochberg FDR correction was set to 0.05. The GO and annotation files were downloaded (http://www.geneontology.org/page/download-ontology) and (http://cvsweb.geneontology.org/cgi-bin/cvsweb.cgi/go/gene-associations/gene_association.goa_human.gz?rev=HEAD), respectively. A non-redundant gene list for the union between the corresponding time points was taken as the background reference. The redundancy of the biological processes was assessed by heatmaps generated in R and GENE-E.

The flowchart below schematically represents the Bioinformatics analysis described above.

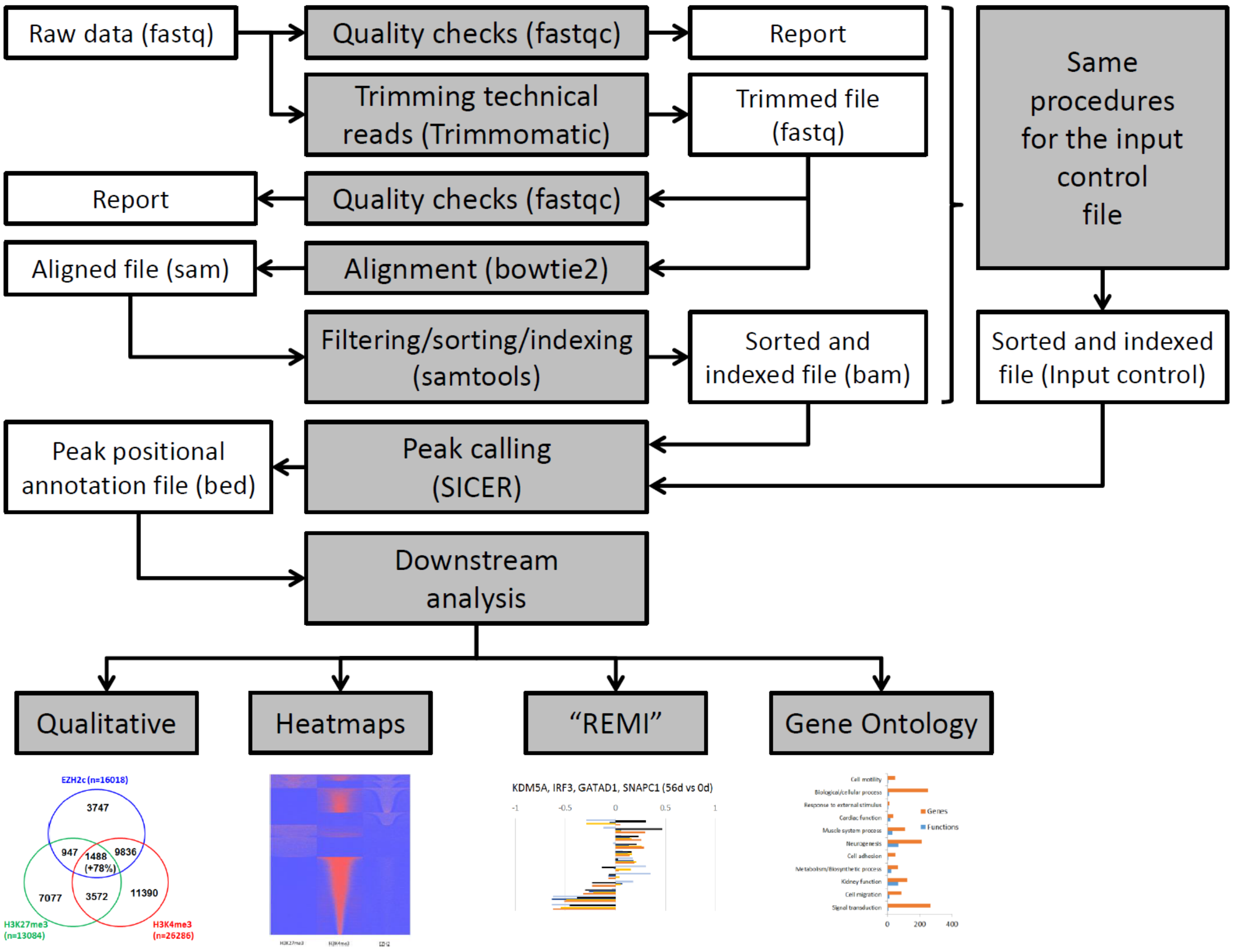

Bioinformatics analysis flowchart. This flowchart describes the tools (grey boxes) and the type of files (white boxes) used in the analysis in this study. Some examples for the downstream analysis were taken from several figures in this paper.

References

1. Bolger AM, Lohse M, Usadel B. Trimmomatic: a flexible trimmer for Illumina sequence data. Bioinformatics (Oxford, England). 2014;30(15):2114-20. doi: 10.1093/bioinformatics/btu170.
2. Langmead B, Salzberg SL. Fast gapped-read alignment with Bowtie 2. Nature methods. 2012;9(4):357-9. doi: 10.1038/nmeth.1923.
3. Zang C, Schones DE, Zeng C, Cui K, Zhao K, Peng W. A clustering approach for identification of enriched domains from histone modification ChIP-Seq data. Bioinformatics (Oxford, England). 2009;25(15):1952-8. doi: 10.1093/bioinformatics/btp340.

4. Quinlan AR, Hall IM. BEDTools: a flexible suite of utilities for comparing genomic features. Bioinformatics (Oxford, England). 2010;26(6):841-2. doi: 10.1093/bioinformatics/btq033.
5. Rendeiro AFF, Schmidl C, Strefford JC, Walewska R, Davis Z, Farlik M, et al. Chromatin accessibility maps of chronic lymphocytic leukaemia identify subtype-specific epigenome signatures and transcription regulatory networks. Nature communications. 2016;7:11938. doi: 10.1038/ncomms11938.
6. Griffon A, Barbier Q, Dalino J, van Helden J, Spicuglia S, Ballester B. Integrative analysis of public ChIP-seq experiments reveals a complex multi-cell regulatory landscape. Nucleic acids research. 2015;43(4). doi: 10.1093/nar/gku1280.
7. Heinz S, Benner C, Spann N, Bertolino E, Lin YC, Laslo P, et al. Simple combinations of lineage-determining transcription factors prime cis-regulatory elements required for macrophage and B cell identities. Molecular cell. 2010;38(4):576-89. doi: 10.1016/j.molcel.2010.05.004.
8. Cline MS, Smoot M, Cerami E, Kuchinsky A, Landys N, Workman C, et al. Integration of biological networks and gene expression data using Cytoscape. Nature protocols. 2007;2(10):2366-82. doi: 10.1038/nprot.2007.324.
9. Maere S, Heymans K, Kuiper M. BiNGO: a Cytoscape plugin to assess overrepresentation of gene ontology categories in biological networks. Bioinformatics (Oxford, England). 2005;21(16):3448-9. doi: 10.1093/bioinformatics/bti551.
