# Supplementary material for: Ibrutinib induces chromatin reorganisation of chronic lymphocytic leukaemia cells
Source: Oncogenesis. 2019 May 10;8(5):32. doi: 10.1038/s41389-019-0142-2 (PMC6510766; doi:10.1038/s41389-019-0142-2)
Supplement: Supplementary file 3 — Supplementary table 1 [file 41389_2019_142_MOESM3_ESM.pdf]

Patient	Treatment Status	Age at entry	BM CLL %	PB CLL x 10^9/L	PB B-cell Ki67 %	TP53 Mutational Status	ATM Mutational Status	IGHV Status	Prog_Cat
N1	Treatment naïve	42.3	92	95	0.7	0	0	Unmutated	Adverse
N2	Treatment naïve	65.6	86	155	3.9	0	0	VH3-21	Adverse
N3	Treatment naïve	64.2	97	49	1.9	96	0	Unmutated	Adverse
N4	Treatment naïve	70.5	92	52	2.35	0	0	mutated	Good
N5	Treatment naïve	71.2	92	55	0.9	91	0	Unmutated	Adverse
N6	Treatment naïve	42.4	93	26	4.85	0	0	mutated	Good
N7	Treatment naïve	55.8	79	43	4.45	0	0	Unmutated	Adverse
N8	Treatment naïve	71.1	91	130	2.05	0	25	Unmutated	Adverse
N9	Treatment naïve	52.2	94	420	1.55	0	96	Unmutated	Adverse
N10	Treatment naïve	55.2	65	0.94	14.65	0	0	mutated	Good
R1	Relapsed/Refractory	52.6	73	18	4.65	0	0	Unmutated	Adverse
R2	Relapsed/Refractory	55.9	84	22	1.15	0	0	Unmutated	Adverse
R3	Relapsed/Refractory	69.4	84	39	4.85	0	0	Unmutated	Adverse
R4	Relapsed/Refractory	62.4	94	243	12.1	0	0	Unmutated	Adverse
R5	Relapsed/Refractory	57	67	46	3.45	0	40	Unmutated	Adverse
R6	Relapsed/Refractory	62.4	88	45	2.45	80	0	Unmutated	Adverse
