# Supplementary material for: Ibrutinib induces chromatin reorganisation of chronic lymphocytic leukaemia cells
Source: Oncogenesis. 2019 May 10;8(5):32. doi: 10.1038/s41389-019-0142-2 (PMC6510766; doi:10.1038/s41389-019-0142-2)
Supplement: Supplementary file 5 — Supplementary table 3 [file 41389_2019_142_MOESM5_ESM.pdf]

Supplementary Table 3 – Primer Sequences

Target	Forward Primer	Reverse Primer	Method
EGR1 -6.1	CCTGGGGTAGGAGCAGAACT	TGTCACACTTTCCGACTGACTT	ChIP
EGR1 -0.85	TACAGTGTCCCAAGAACCAAGT	CGATCTATGGCACGGTGTCTTT	ChIP
EGR1 -0.1	GCCATATTAGGGCTTCCTGCTT	GATCCGCCTCTATTTGAAGGGT	ChIP
EGR1 +2.2	CTGCGACATCTGTGGAAGAAAG	GCCGCAAGTGGATCTTGGTAT	ChIP
DUPS2 +2.4	GGAAGTGATGGGTGTGTCATGT	AAATAATTTTCCAGCGCCAGCA	ChIP
MYC +0.1	TGCCTCTCGCTGGAATTACTAC	GAGGGATCGCGCTGAGTATAAA	ChIP
DUSP4 +0.2	GACCGGCAAAAATACACGGGA	GGAGGAGAGTGTGTTTACGAGA	ChIP
CTCF1	GGCCCAGGACTCCACGTTCAGA	GCCCTCTGGTGTTTGGCAGCAA	ChIP
CTCF2	CACCCAGCAGAGGGCCCAGATA	CCCTTCGCCTTCTCTCCAGCCA	ChIP
CXCR4 -0.4	TCCAGACCTGGGAATGCTAC	GTTGGAAGCTTGGCCCTACT	ChIP
CXCR4 -14	AGGCAGCCCTAAAGGGAATA	TGTCCCAAGCTCCACTCTCT	ChIP
CXCR4 -18	ATAGGGCGGTGCAGTACAGT	ACCCTGGGCTTTAGTTTGCT	ChIP
CXCR4 -261	GGGCAGTGGCTTTCTAAGTG	CTAGCTGGGAGCCAAGAATG	ChIP
CXCX4	GCACATCATGGTTGGCCTTATC	GAGTGTGACAGCTTGGAGATGA	RT-qPCR
RAC1	ACCGGTGAATCTGGGCTTATG	AAGAACACATCTGTTTGCGGA	RT-qPCR
